# The Chameleon Strategy—A Recipe for Effective Ligand Screening for Viral Targets Based on Four Novel Structure–Binding Strength Indices

**DOI:** 10.3390/v16071073

**Published:** 2024-07-03

**Authors:** Magdalena Latosińska, Jolanta Natalia Latosińska

**Affiliations:** Faculty of Physics, Adam Mickiewicz University, Uniwersytetu Poznańskiego 2, 61-614 Poznań, Poland

**Keywords:** chameleon strategy, molecular chameleons, RNA viruses, SARS, structure–binding affinity index, structure hydrogen bond index, structure steric effect index, structure protein–ligand index, drug design, novel approach, molecular docking, molecular dynamics simulations

## Abstract

The RNA viruses SARS-CoV, SARS-CoV-2 and MERS-CoV encode the non-structural Nsp16 (2′-O-methyltransferase) that catalyzes the transfer of a methyl group from S-adenosylmethionine (SAM) to the first ribonucleotide in mRNA. Recently, it has been found that breaking the bond between Nsp16 and SAM substrate results in the cessation of mRNA virus replication. To date, only a limited number of such inhibitors have been identified, which can be attributed to a lack of an effective “recipe”. The aim of our study was to propose and verify a rapid and effective screening protocol dedicated to such purposes. We proposed four new indices describing structure-binding strength (structure–binding affinity, structure–hydrogen bonding, structure–steric and structure–protein–ligand indices) were then applied and shown to be extremely helpful in determining the degree of increase or decrease in binding affinity in response to a relatively small change in the ligand structure. After initial pre-selection, based on similarity to SAM, we limited the study to 967 compounds, so-called molecular chameleons. They were then docked in the Nsp16 protein pocket, and 10 candidate ligands were selected using the novel structure-binding affinity index. Subsequently the selected 10 candidate ligands and 8 known inhibitors and were docked to Nsp16 pockets from SARS-CoV-2, MERS-CoV and SARS-CoV. Based on the four new indices, the best ligands were selected and a new one was designed by tuning them. Finally, ADMET profiling and molecular dynamics simulations were performed for the best ligands. The new structure-binding strength indices can be successfully applied not only to screen and tune ligands, but also to determine the effectiveness of the ligand in response to changes in the target viral entity, which is particularly useful for assessing drug effectiveness in the case of alterations in viral proteins. The developed approach, the so-called chameleon strategy, has the capacity to introduce a novel universal paradigm to the field of drugs design, including RNA antivirals.

## 1. Introduction

The chameleon strategy is the ability to adapt to any environment and circumstances and thus to changing conditions. Molecules that exhibit certain properties that allow them to adapt to an environment or mimic naturally occurring moieties can be called “molecular chameleons”. New broadly acting drugs with inhibitor activity against SARS should be able to use this strategy and easily adapt to mutating viral structures as well as different types of viruses. It seems that the ideal inhibitor should act like a Trojan horse: unnoticeable to the host and virus, harmless to the host but harmful to the virus. Crypsis helps to avoid detection by the host and can be realized using shapes, conformation, polarization, etc., comprising total binding ability. Therefore, this branch of research represents a significant area of current interest in the field of drug design targeting human RNA viruses. Our goal was to determine factors which should be taken into account when designing them and approaches that may be helpful and accelerate the whole process.

### 1.1. The State of the Art

So far, seven different strains of human coronaviruses have been identified, namely α-CoV (HCov-229, 1960s [1] and HCov-NL63, 2004 [2]) and β-CoV (HCov-OC43, 1960s [3]; HCov-HKU1, 2005 [4]; SARS-CoV, 2002–2003 [5]; MERS-CoV, 2012 [6] and SARS-CoV-2, 2019 [7]); see Table 1. Among them, SARS-CoV, MERS-CoV and SARS-CoV-2 are highly aggressive [8]. The Global Initiative on Sharing All Influenza Data (GISAID) classifies SARS-CoV and SARS-CoV-2 in Clade I, lineage B, while MERS-CoV is in Clade I, lineage C. At the whole genome level, SARS-CoV-2 shares approximately 79% sequence similarity with SARS-CoV and approximately 50% sequence similarity with MERS-CoV [9]. All SARS-CoV-2 variants of concern share 97% sequence similarity. 

However, regardless of the different level of sequence conservation, their genomic arrangement is generally similar [10,11]. Their core is primarily composed of four structural proteins: nucleocapsid protein (N), spike protein(S), envelope protein (E), membrane protein (M), 16 non-structural (Nsps) and 9 accessory (ORFs) proteins [12,13,14]. Each CoV is an enveloped virus with numerous club-like spikes protruding from the surface, having an unsegmented, single-stranded, positive-sense RNA (+ssRNA) genome with a 5′-cap and 3′-poly(A) tail. Both allows genome to act as functional mRNA for translational synthesis of the replicase polyproteins [15]. The 5′-cap, essential for stability and function, serves to protect mRNA molecules from degradation to recruit processing factors and initiate translation [16]. Furthermore, the 5′ end RNA cap is analogous to human mRNA, as both contain C2′-O-methylribosyladenine and N-methylated guanosine triphosphate. This enables the virus to utilize the host cell for protein production and protects the viral mRNA from recognition as foreign by cellular sensors, thereby evading the host cell’s immune system. Two-thirds of the viral genome is occupied by a replicase gene consisting of two open reading frames (ORFs), ORF 1a and ORF1ab, which encode the non-structural proteins (Nsps), the so-called pp1a and pp1ab polyproteins, respectively. The two non-structural proteins pp1a and pp1ab include Nsp1 to Nsp11 and Nsp12 to Nsp16, respectively. The 3′UTR-poly (A) tail follows the translation termination codon of mRNA, with accessory genes such as ORF3a, ORF3d, ORF6, ORF7a, ORF7b, ORF8, ORF9b, ORF14 and ORF10 [17] interspersed among the structural genes preceding the 3′ end of the viral RNA genome. These structural genes encode nine accessory proteins and are considered important virulence factors involved in various pathogenesis mechanisms during SARS-CoV-2 infection [18,19]. The capsid, a shell of a virus enclosing its genetic material, formed from the nucleocapsid protein (N), is located outside the +ssRNA genome. The genome is additionally covered by an envelope that is mainly associated with three structural proteins: membrane (M), spike (S) and envelope (E). The S protein, responsible for binding to the host entry receptor—angiotensin-converting enzyme-2 (ACE-2) [20,21] or dipeptidyl peptidase 4 (DPP4) [22]—and for cell attachment and entry to the host cell (membrane fusion) forms homotrimers that can protrude from the viral surface and act as a direct target of the host’s immune response [23]. The E protein, as a viral ion channel (viroporin), is responsible for ion conduction. It is considered the smallest of the main structural proteins, but an important element of virulence in SARS-CoV-2 [24]. E-protein plays a vital role because it assists in the insertion of the virus into the host cell and controls the pathogenicity of the virus. The M protein, the most common viral structural protein, is the main driver of viral assembly and membrane construction [25]. It is a dominant structural protein that can combine with other structural proteins such as S and E proteins [26]. Thus any mutation in the M protein is expected to have a significant impact on the interactions with the infected cell [27]. Recently, there has been a notable increase in optimism regarding the potential of nonstructural proteins (Nsps) as therapeutic targets. This is attributed, among other factors, to the observation that Nsp proteins are highly conserved regions in the genomes of SARS-CoV, MERS-CoV and SARS-CoV-2. Most Nsps participate mainly in the virus replication, immune response and modulation (see Table 2), but also have specific roles in the replication of CoVs [28].

**Figure 1:**
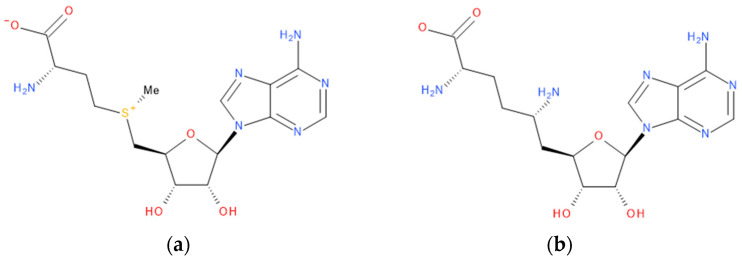
The structures of (**a**) S-adenosylmethionine (SAM, AdoMet)—essential for coronavirus replication and (**b**) sinefungin—the known Nsp16 inhibitor.

Immediately after infection, the resulting viral RNA is translated to produce a polyprotein, which is then cleaved to produce 16 non-structural proteins (Nsps) involved in genomic/subgenomic RNA transcription [29]. The replication and transcription complex, which participates in genome replication and transcription of mRNA, includes nonstructural proteins Nsp12 (RNA-dependent RNA polymerase, RdRp), Nsp7 and Nsp8. However, these proteins form only the so-called RdRp core complex, which binds several additional nonstructural proteins Nsp9, Nsp10, Nsp13, Nsp14 and Nsp16. CoVs adopt different strategies to escape the multifaceted immunity of infected cells by using Nsps. Their caps are composed of Nsp10 (cofactor), Nsp13 (5′-triphosphatase), Nsp14 (N7-methyltransferase) and Nsp16 (2′-O-methyltransferase). Nsp13, guanylate kinase (GK1), Nsp14 and Nsp16 participate in enzymatic reactions responsible for the RNA cap installation, which is crucial for virus stability. Melanoma differentiation-associated protein 5 (MDA5), a cytoplasmic receptor playing a role of intracellular sensor of viral RNA, triggers the innate immune response. Nsp16 (2′-O-Ribose-methyltransferase), active only in the presence of Nsp10 playing a catalytic role, protects RNA viruses from identification by MDA5 [19]. Nsp16 combined with Nsp10 is responsible for efficient viral mRNA cap 2′O-methylation, which allows the virus to mimic the host mRNA and enables it to replicate efficiently without activating immune defense. The enzymatic activity exhibited by the heterodimer comprising Nsp10 and Nsp16 is essential for the methylation of the 5′ end of the RNA transcript, specifically the viral cap. S-Adenosylmethionine (SAM, AdoMet), Figure 1a, formed from adenosine triphosphate (ATP) and methionine by methionine adenosyltransferase, is a methyl group donor that moves to the 2′-O position of the ribose in the Cap-0 [30]. 

Methylation of the RNA cap shields viral RNA from MDA5 recognition [19] and helps deceive innate human immune mechanisms, which recognize the viral RNA and activate the immune response. Thus, Nsp16, an S-adenosyl-L-methionine (SAM, SAM-e)-dependent methyltransferase, is essential for coronavirus replication [30,31]. It has been demonstrated that the disruption of the binding between Nsp16 and the substrate SAM inhibits its activity, which subsequently results in a cessation of viral replication. The replication of SARS-CoV, which lacks functional Nsp16, is significantly reduced in vitro and in mouse models [32]. Consequently, the SAM binding site is regarded as a highly attractive target. The high conservation of the MTase active site across SARS-CoV-2, SARS-CoV and MERS-CoV implies that Nsp16 inhibitors have the potential to target multiple RNA viruses. The Nsp16 from the three coronaviruses shows significant conservation exceeding 65%, with SARS-CoV and SARS-CoV-2 showing 93% sequence similarity, SARS-CoV-2 and MERS-CoV 66% and SARS-CoV and MERS 65%. Given that SARS-CoV-2 variants of concern share 97% sequence similarity, higher than SARS-CoV or MERS, conserved SAM binding pocket seems a very promising target. The conservation of Nsp10–Nsp16 is an indication that broad-spectrum inhibitors may be developable. Therefore, an approach targeting Nsp16 seems to be future-proof and viable for combating existing and future CoVs, as well as other RNA viruses that use the 2′O-MTase mechanism for capping. The advantage of inhibiting Nsp16 is that it effectively hinders the process of viral replication while enhancing the immune response. 

Known Nsp16 inhibitors include S-adenosyl-L-homocysteine analogs: sinefungin [33,34], Figure 1b; tubercidin [35] and its derivative, SS148 [36,37]; toyocamycin; sangivamycin; 5-iodotubercidin (Kremling et al., Protein Data Bank (PDB), accessed on 10 February 2024); the adenine derivative WZ16 [36,37] and adenosine derivative W08 (Kremling et al., Protein Data Bank (PDB), accessed on 10 February 2024); and adenosylhomocysteinase (AHCY) [35]. Sinefungin shows low inhibitory effect in cell culture-based assays [38,39,40]. Tubercidin has broad antiviral effects but is poorly selective and cytotoxic. However, its derivatives have better activity profiles [36,37]. Toyocamycin has a better profile than sangivamycin and 5-iodotubercidin, but its efficiency is not known. As Rosas-Lemus [38] suggests, the greatest hopes for Nsp16 inhibitors are associated with analogues of SAM and its demethylated form, S-Adenosyl-L-homocysteine (SAH). SAH, the product of all adenosylmethionine (SAM)-dependent biological transmethylations, is broken down to homocysteine and adenosine by S-adenosylhomocysteine hydrolase enzyme. 

### 1.2. The Underlying Motivation and Approach Concept

Identification of effective inhibitors for viral proteins represents a significant challenge in the field of antiviral research. However, the entire process of finding candidate ligands and then leads is extremely time-consuming and, even in silico, expensive. In the context outlined above, the objective of our paper was to propose an approach helping to identify ligand sufficiently similar to SAM and flexible enough to form a strong binding with Nsp16. The challenge was to discover a method to achieve it quickly. 

Typically, a pre-search is conducted using already known ligands, 2D structure template or principal component analysis [41,42,43,44,45,46,47,48], but SAM and SAH are extremely flexible structures; thus the success of this strategy can be limited. A combined data mining using the SAM/SAH structure as a template and 3D conformational similarity as an acceptance/rejection criterion helped to identify 967 candidate ligands with relatively high structural 3D similarity to SAM, the so-called molecular chameleons. Subsequent molecular docking reduces the chemical space in the search for ligands that are able bind to a target protein [49,50,51,52,53,54]. However, the decision process based on these data is not easy. The classical analysis of docking results is limited to the docking scores or binding affinity (BA), which describe the strength of the protein–ligand interactions. They are generally not used for bulk screening of ligands. In light of the aforementioned considerations, we have put forth a set of dedicated indices, comprising a finite number of instructions and exhibiting low computational complexity. Furthermore, we have proposed an approach based on these indices that is expected to markedly enhance the decision-making process. Four structure–interaction strength indices: structure-binding affinity index (SBAI), structure hydrogen bonding index (SHBI), structure steric effect index (SSEI) and structure protein ligand index (SPLI) reveal the extent to which binding affinity, hydrogen bondings, steric effect or protein–ligand interaction strength are gained or lost in response to the relatively small changes in the ligand structure. Each of these indices elucidate the strength of binding between protein and ligand in relation to structure. The absolute value of SBAI may be employed as a means of identifying potential ligand candidates. In order to facilitate further analysis of the candidate ligands all four indices are utilized. These indices, combined with in-depth analysis of protein–ligand binding modes based on mathematical metrics and heatmaps, constitute an approach we call the chameleon strategy, which allows us to fine-tune candidate ligands and find promising new ones. The developed approach supports effective screening and the search for new directions for improving inhibitors.

The usefulness of the developed chameleon strategy was verified on the SAM analogues, molecular chameleons. As demonstrated below, the chameleon strategy can be successfully applied to ligand screening and tuning, but also for determining the efficacy of the ligand in response to alterations in the targeted viral entity. The approach proposed in this paper is a continuation of our ongoing research [51,52,53,54,55] towards developing new techniques for searching and analyzing known inhibitors as well as candidates for them. 

## 2. Materials and Methods

### 2.1. Ligand Selection

In the first step, data mining using the SAM structure as a template and 3D similarity (so-called conformational similarity) as an acceptance/rejection criterion was used. The simplified molecular-input line-entry system SMILES, which obtains a string when traversing the tree of a chemical graph representing the spanning tree of a chemical graph and improves ligand preparation workflows, was used as a molecular fingerprint. In its extended form, it provides a linear notation describing chemical structure, including composition, bond order, aromaticity, branching and stereochemistry. The pre-selection of structurally similar compounds from the whole set of a few thousand structures somehow related to SAM was based on the Tanimoto [56,57] coefficient, which quantifies the degree of similarity between the fingerprints. Replacement of the sulfur by another atom (e.g., N or O) was assumed to be permissible when searching for structures containing SAM as a substructure and the 90% similarity criterion was employed. In this manner, a selection of a set of structurally similar 967 ligands, the so-called molecular chameleons, was made. Subsequently, their structures were optimized at the M062X/6-31G(d,p) level of theory. The calculations were carried out using Gaussian 16 rev. C01 [58]. As we have shown previously, M062X provides a reliable electron density distribution in single molecules and cluster systems with non-covalent interactions [51,52,53,54,55].

### 2.2. Target Selection

Nearly 5000 experimental structures of SARS-CoV-2, SARS-CoV-1 and MERS-CoV were deposited in the Protein Data Bank (PDB) (see Table 3) including 16 protein structures, two functional domain structures of nucleocapsid (N) protein and dozens of complexes. 

Although much research has focused on the spike protein S and critical structural proteins M and E, the crystal structures of the non-structural proteins are also available. The co-crystal structures with S-adenosylmethionine (SAM, AdoMet), necessary for coronavirus replication, and sinefungin, a known Nsp16 inhibitor, were searched for at the best available resolutions. The crystal structures of the Nsp10-Nsp16 heterodimers from MERS-CoV (5YN6 and 5YNB [59]), SARS-CoV (3R24 [60] and 2XYR [30]) and SARS-CoV-2 (6W4H [38], 6WKQ [38] and 6XKM [61]) were retrieved from the Protein databank PDB database (https://www.rcsb.org, accessed on 20 March 2024).

### 2.3. Molecular Docking

In order to achieve conclusive outcomes, it is necessary to possess not only a trustworthy structure of the ligand and protein, but also to employ an appropriate molecular docking (MD) protocol. MD is used to model the protein–ligand poses and provides a basis for the further analysis of protein–ligand interactions and predicting ligand activity [62,63,64,65].

The initial molecular docking attempt was conducted using automated docking tools AutoDock ver. 4.2.6 [66] and AutoDock Vina ver. 1.2.3 [67]. The receptor and ligand structures were converted to the .pdbqt format using MGLTools ver. 1.5.7. Since the semi-empirical docking does not always give reliable results, it was verified by re-docking using the empirical one, i.e., genetic evolutionary method for molecular DOCKing (GEMDOCK) [68]. GEMDOCK scoring employs a different evolution operator and piecewise potential energy integrated into the differential evolutionary algorithm during the docking process. The advantage of GEMDOCK is the use of empirical scoring, which includes pharmacophore potential, and the accuracy of the results obtained is comparable to that of results from AutoDock 4 or AutoDock Vina. Prior to docking, the native ligand that co-crystallized with Nsp-10-Nsp16 heterodimer and water molecules were removed from the structure, and the protein protonation state was corrected. To check the correctness of the docking protocol, re-docking of native ligand was performed. The root means square deviation (RMSD) of the pose from its conformation in the parent structure, not exceeding 2–3 Å, was employed as a metric for the validity of the docking protocol. The candidate ligands were docked in the protein structure using two techniques: template docking and docking with a defined search space (the grid box of size 9–15 Å was centered on the active site). One of the most important limitations in molecular docking is the neglect of protein flexibility. To address this problem, a certain degree of flexibility was assumed for selected residues within the active site. After docking, the optimal poses that provide the most robust stabilization of the protein–ligand complex were selected for further analysis. 

The Gehlhaar model [69] with original empirical parameterization was used for the estimation of the binding affinity. The final 2D and 3D visualizations of the binding modes were made using PoseEdit [70] and VMD [71].

### 2.4. Molecular Dynamics Simulation (MDS)

Molecular dynamics simulations (MS), widely used to refine structural models, were performed using the coarse grain technique [72,73], which is a modification of the classical molecular dynamics approach (MD). The coarse-grained approach [74] can be described as a process of renormalization of the interactions into new representation with simultaneous dimensionality reduction. The equivalent particles concept helps to reduce the computational complexity of the simulation. A total of 50,000 models can be produced in each MS task, with 2% selected for subsequent comparison studies.

RMSF (root mean square fluctuation), which indicates the differences in the positions of entire structures over time, was used to assess the individual flexibility of the residuals. RMSF is defined as

(1) $$RMSF=\sqrt{\sum_{i}{<\left(x_{i}-{<x}_{i}>\right)}^{2}>}$$

where x_i_—the coordinates of particle i and ⟨x_i_⟩ the ensemble average position of i. Plotting the RMSF per residue versus residue number enabled the identification of the amino acids in the protein that contribute most to molecular motion. It is worth noting that the backbone or alpha-carbon atoms are usually more indicative of conformational changes than the more flexible side chains.

### 2.5. Numerical Techniques

#### 2.5.1. Root Mean Square Deviation of the Binding Mode

The average deviation between the binding modes was calculated using the newly defined quantity. Root mean square deviation of the binding modes (RMSD_BM) [51] was calculated as follows:

(2) $$RMSD\text{\_}BM(P,Q)=\sqrt{\frac{1}{n}\sum_{i}{\left(p_{i}-q_{i}\right)}^{2}}$$

where p_i_ and q_i_ are the binding interactions in each structure and P = {p_i_} and Q = {q_i_}.

#### 2.5.2. Heatmaps 

Heatmaps, a two-dimensional data visualization technique in which the magnitude of individual values in a dataset are color-coded, was used to help capture the most relevant data. We applied gridded color-coded heatmaps to visualize the following:

- the protein–ligand binding modes,
- the new indices (defined below),
- the normalized B-factors (the distribution of residue harmonic oscillations),
- the root-mean-square-fluctuation (RMSF).

Native and docking-derived complexes were compared using a red–yellow–blue scheme, with dark red and dark blue indicating strong and weak interactions, respectively.

#### 2.5.3. Structure–Binding Strength Indices 

To assess differences in the protein–ligand binding affinity, we defined in this paper a set of new parameters, the so-called structure-binding strength indices (SBSI), as follows:

(a) Structure-binding affinity index, *SBAI*:
  (3) $$SBAI=\frac{{BA}_{reference}-{BA}_{ligand}}{1-s}=\frac{d_{BA}}{1-s}$$
  which measure the difference in the protein–ligand binding affinity between the candidate and reference ligands (numerator) with respect to their structural similarity (denominator),
(b) Structure–hydrogen bond index, *SHBI*:
  (4) $$SHBI=\frac{{HB}_{reference}-{HB}_{ligand}}{1-s}=\frac{d_{HB}}{1-s}$$
  which measures the difference in the energy of hydrogen bonds (connecting the protein with the ligand) in the complex between the selected ligand and the reference compound (numerator) with respect to their structural dissimilarity, 1 − s, (denominator),
(c) Structure–steric effect index, SSEI:
  (5) $$SSEI=\frac{{SE}_{reference\;}-{SE}_{ligand}}{1-s}=\frac{d_{SE}}{1-s\;}$$
  which measures the difference in the energy of steric effects between the complex with the selected ligand and the reference (numerator) with respect to their structural dissimilarity, 1 − s, (denominator),
(d) Structure–protein–ligand index, *SPLI*:
  (6) $$SPLI=\frac{{PL}_{reference}-{\;PL}_{ligand}}{1-s}=\frac{d_{PL}}{1-s}$$
  which measures the difference in the strength of the protein–ligand interactions between the ligand and reference (numerator) with respect to their structural dissimilarity, 1 − s, (denominator).

d_i_, i ∈ {BA, HB, SE, PL} in the numerator is derived from the molecular docking results, while s in the denominator is the 3D pharmacophore similarity.

**Figure 2:**
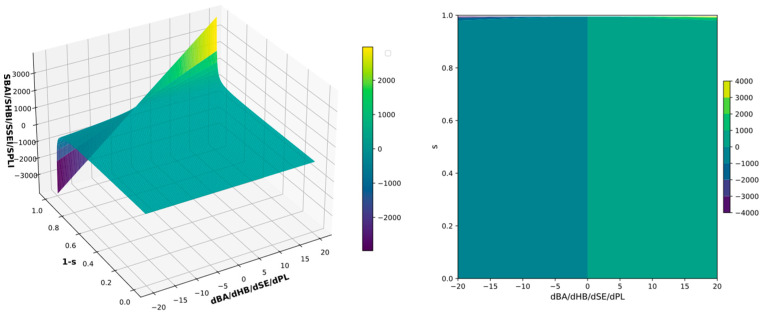
The 3D surface (**left**) and contour (**right**) plots of structure–binding strength indices (SBSI). The d_i_ independent variable was truncated to the range from −20 to 20; the SBSI(d_i_, s) was truncated for visualization purposes.

Mathematically speaking, all four indices (Equations (3)–(6)) are 2D functions that change rapidly as the values of the independent variables’ d_BA_/d_HB_/d_SE_/d_PL_ and s change. A uniform mathematical function SBSI(d_i_, s) = d_i_/(1 − s), where i ∈ {BA, HB, SE, PL} can be used to describe all of them. Its domain is D_SBSI_ = {{d_i_, s}: d_i_ ∈ R, s ∈ <0, 1>}. Nevertheless, the d_i_ variable is only theoretically capable of reaching infinite value; consequently, it can be truncated to a reasonable range from −20 to 20. The codomain, or range, is as follows: D’_SBSI_ = R. The 3D surface plot and the contour plot show SBSI(d_i_, s) variability; see Figure 2.

The smaller the structural difference between the ligand and reference, the higher the index values; for the ligand identical to the reference, indices are infinitely large; see Figure 2. The function SBSI(d_i_, s) is very sensitive to small structural changes, but poorly sensitive to large ones. The specific construction of the function helps differentiate whether the studied parameter i ∈ {BA, HB, SE, PL} is slightly better or worse for the studied ligand in comparison to the reference. 

SBSI indices are a measure of the extent to which binding affinity/hydrogen bonds/steric effect/protein–ligand energy are gained or lost in response to the relatively small changes in the ligand’s structure. The heatmap generated using SBSI indices enables the identification of the most promising ligand.

### 2.6. ADMET Drug-Likeness Evaluation

The in silico ADMET (Absorption, Distribution, Metabolism, Excretion, and Toxicity) drug-likeness evaluation was performed using the SwissADME developed by the Swiss Institute of Bioinformatics [75] and ADMET 2.0 [76]. Drug-likeness was tested according Lipinski, Veber and Egan’s rules of 5 (RO5). The Abbot Bioavailability scores were computed to predict the probability of a compound to have at least 10% oral bioavailability. Lipophilicity was predicted with iLOGP, XLOGP3, WLOGP, MLOGP and SILICOS-IT models from which a consensus log Po/w was determined [75]. The solubility (log S) of the ligands was predicted using SILICOS-IT model. The synthetic availability (SA) was determined based on the frequency of molecular fragments in “truly” obtainable molecules. The mutagenicity/carcinogenicity risk scores, Caco-2, Madin–Darby Canine Kidney (MDCK) intestinal cell permeability and QED, a complex measure of attractiveness, were calculated on the basis of drug-likeness parameters: molecular weight (MW), lipophilicity (log P), N_HBA_ (number of HB acceptors), N_HBD (_number of HB donors), PSA, N_rotb_, the number of aromatic rings (N_Ar_), molecular polar surface area (PSA) and the number of alerts for undesirable functional groups, were predicted using models implemented in ADMET2.0 [76]. 

## 3. Results and Discussion

**Figure 3:**
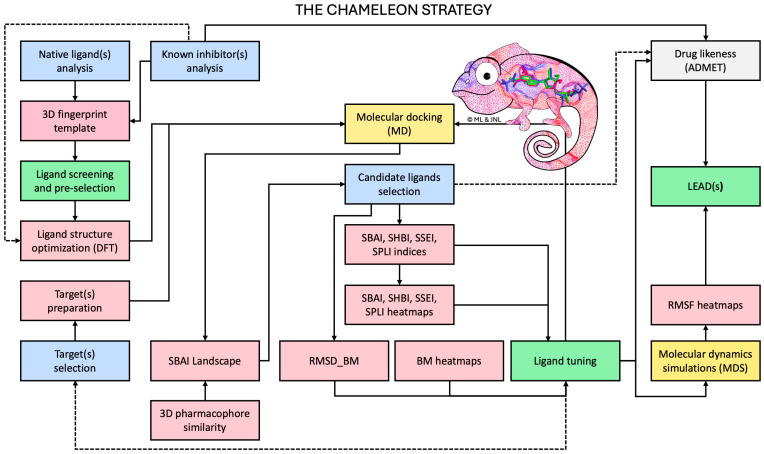
The chameleon strategy flowchart. The blue and green boxes represent steps, yellow, grey and pink boxes indicate the methods, the solid and dashed line shows fundamental and optional paths, respectively.

The workflow diagram illustrating so called chameleon strategy, which was proposed in this paper, is shown in Figure 3.

### 3.1. Step I: The Analysis of the Targets, Native Ligand and Known Inhibitor

#### 3.1.1. The Targets

In the first step (see Figure 2) the entire target sequences and binding modes of the native ligand SAM and known inhibitor, sinefungin, in co-crystals were analyzed. The crystal structures of the Nsp10-Nsp16 heterodimers from MERS-CoV (5YN6 and 5YNB [59]), SARS-CoV (3R24 [60] and 2XYR [30]) and SARS-CoV-2 (6W4H [38], 6WKQ [38] and 6XKM [61]) were compared. 

**Figure 4:**
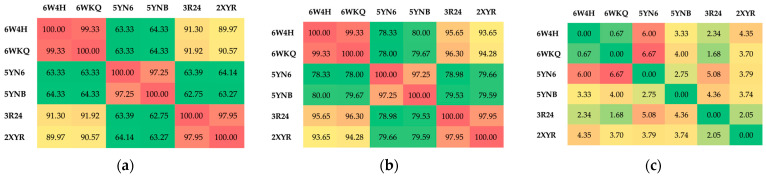
Protein alignment across the Nsp16 structures (**a**) identity—left, (**b**) similarity—middle and (**c**) gaps—right in the protein sequences. The most similar protein pairs in terms of identity and similarity are marked in red and the least similar in green. The largest number of gaps are marked in red; the smallest number is marked in green. The EBLOSUM62 matrix with gap penalty (GOP) = 2.0 and gap extension penalty (GEP) = 2.0 was used.

Protein alignment provides a global comparison of the percentage of identity (the percentage of identical residues), similarity (likelihood for sequence homology) and gaps (residues deleted) across the entire sequence, Figure 4. The Needleman–Wunsch algorithm [77] was used, and the scores were computed using the most efficient EBLOSUM62 matrix [78], with the gap opening penalty (GOP) = 2.0 and gap extension penalty (GEP) = 2.0. The result is visualized using heatmaps. 

The Nsp16 of the same viruses but with a different ligand show the greatest similarity and identity as expected. The least similarity/identity is shown by the following pairs (5YN6, 6W4H), (5YNB, 6WKQ), (5YNB, 6W4H) and (5YNB, 6WKQ). Thus, Nsp16 from SARS-CoV-2 and SARS-CoV reveals a higher level of conservation than that from MERS-CoV. The largest gaps occur in pairs (5YN6, 6W4H) and (5YN6, 6WKQ). 

**Figure 5:**
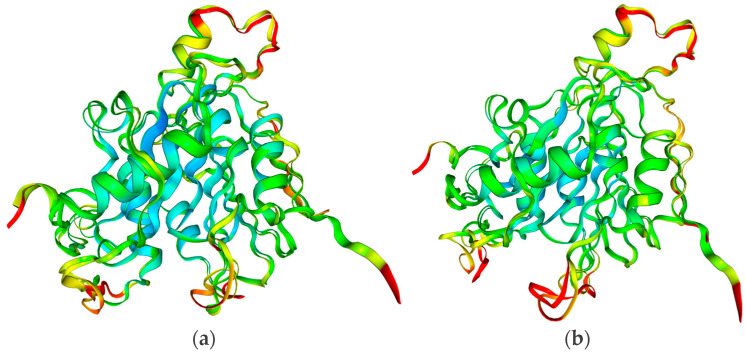
The superimposed structures of Nsp16 (**a**) SARS-CoV-2 (6W4H) vs. MERS-CoV (5YN6) and (**b**) SARS-CoV-2 (6W4H) vs. SARS-CoV (3R24) with the rigid areas marked in red (red ∆B = +4, blue ∆B = −2). The region of increased rigidity is located near Glu7062 (6W4H) and TYR30, Thr140 and Gln266 (6XKM), Arg38 (5YN6) and TYR 30 and Lys141 (3R24 and 2XYR).

The geometry of the Nsp16 chain A also reveals a high degree of resemblance; see Table 4 and Figure 5. The root mean square deviation (RMSD) between the pairs is low, with (6W4H, 6WKQ), (5YN6, 5YNB) and (3R24, 2XYR) differing by two, eight and six residues, respectively. 

**Figure 6:**
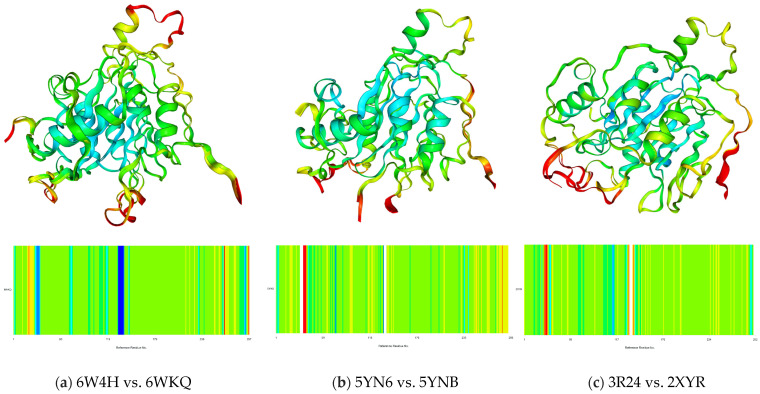
The superimposed structures of Nsp16 (**a**) SARS-CoV-2 (6W4H vs. 6WKQ), (**b**) SARS-CoV (3R24 vs. 2XYR) and (**c**) MERS-CoV (5YN6 vs. 5YNB) with the rigid areas marked in red (red ∆B = +4, blue ∆B = −2). The region of increased rigidity is located near Glu7062 (6W4H) and TYR30, THR140 and GLN266 (6XKM), Arg38 (5YN6) and TYR 30 and Lys141 (3R24 and 2XYR). The white bands serve to indicate the gaps.

The SAM-engaging residues are indeed conserved in RNA viruses (SARS-CoV-2, SARS-CoV and MERS-CoV) (Figure 6) which is in line with [79,80]. 

The superimposed structures of Nsp16 from SARS-CoV 2 (6W4H), MERS-CoV (5YN6) and SARS-CoV (3R24) with the areas of increased rigidity marked in red are shown in Figure 5 and Figure 6. The replacement of SAM with sinefungin results in an increase in the stiffness in MERS-CoV and SARS-CoV, but in a decrease in SARS-CoV-2, albeit at sites far from the binding site; see Figure 6. 

The size, shape and hydrophobicity of the pocket are important factors in predicting protein–ligand binding. The parameters describing the anatomy of protein pockets are listed in Table 5. 

The surface–volume ratio lies within the range of 1.44 to 1.59. Sinefungin forms more hydrogen bonds than SAM; however, both accept rather than donate protons to hydrogen bonds. The average hydrophobicity of the pocket is relatively constant and slightly lower for SARS-CoV-2. The analysis of the details in the surface hydrophobicity help to identify regions of the pocket most likely to interact with a binding ligand. In general, the highly hydrophobic residues bind the non-polar adenine moiety and the less hydrophobic non-polar methionine moiety of the native ligand.

#### 3.1.2. The Native Ligand and Known Inhibitor Binding Mode 

As follows from the Tables S1–S7 and Figure 5 and Figure 6, the overall pattern of binding within the pairs of Nsp16 is similar and protein–ligand energy should depend on the contribution of the hydrogen bonds. To assess the potential influence of these differences on binding, we conducted further detailed analysis of the protein–ligand binding modes. 

In the 6W4H [38] co-crystal, the adenosine part of SAM is hydrogen-bonded to LEU6898, CYS6913 and ASP6912, the glycone part is hydrogen-bonded to GLY6871, ASN6899 and TYR6930, and the methionine part is hydrogen-bonded to ASN6841, GLY6869 and GLY6879; see Table S1. Furthermore, each ligand moiety is stabilized by three water bridges with GLY6869, Ser6872 and ASP6897. Additionally, a long salt bridge of 4.93 Å binds ASP6928 to sulfur.

In the 6XKM [61] co-crystal, the adenine part of SAM is hydrogen-bonded to As114 and CYS115, the glycone part is hydrogen-bonded to TYR132, LEU100, ASN101 and GLY73, and the methionine part is hydrogen-bonded to ASN43, Ty47, GLY71, ASP130 and GLY81; see Table S2. Each ligand moiety is further stabilized by four water bridges with GLY71, Ala72, Ser 74 and ASP99. Additionally, a long salt bridge of 4.88 A binds ASP130 to sulfur.

In the 6WKQ [38] co-crystal the hydrogen bonding pattern is similar to that described above. The adenine moiety of sinefungin is hydrogen-bonded to ASP6912 and CYS6913, the glycone moiety to GLY6871, LEU6898 and TYR6930 and the methionine moiety to ASN6841, TYR6845, GLY6869 and ASP6928; see Table S3. Each ligand moiety is stabilized by ten water bridges with GLY6869, ASP6873, ASP6897, ASN6899, ASP6928, PHE6947 and LYS6968.

In the 5YN6 co-crystal [59], the hydrogen bonding pattern is close to that described above. Th adenine moiety of SAM binds to LEU100, CYS15 and ASP114, the glycone moiety to GLY73, ASN101 and TYR132 and the methionine moiety to ASN43, TYR47 and GLY71; see Table S4. Additionally, a long salt bridge of 4.90 A binds ASP130 to sulfur. The only difference between SARS-CoV-2 and MERS is that methionine binds to TYR instead of GLY and additionally to ASP.

In the 5YNB co-crystal [59], the hydrogen bonding pattern is highly similar to that described above. They adenine moiety of sinefungin binds to LEU100, CYS15 and ASP114, the glycone moiety to GLY73 and TYR132 and the methionine moiety to ASN43, TYR47, GLY71, GLY81 and ASP130; see Table S5.

In the 3R24 co-crystal [60], the adenine of SAM binds with hydrogen bonds to LEU100, CYS115 and ASP114, the glycone moiety to GLY73 and TYR132 and the methionine moiety to TYR47, GLY71, GLY81 and TYR132; see Table S6. Additionally, a long salt bridge of 5.08 A binds ASP130 to sulfur. The only difference between SARS-2 and SARS-CoV binding mode is that methionine forms 4 hydrogen bonds instead of 3, while glycone forms only 2 instead of 3. Structure is additionally stabilized by seven water bridges binding the ligand to GLY71, ALA72, SER74, ASP99, ASN101 and PHE149.

In the 2XYR co-crystal [30], the adenine moiety of sinefungin binds to LEU100, CYS115 and ASP114 using hydrogen bonds, the glycone moiety to GLY73, ASN101 and TYR132 and the methionine moiety to ASN43, GLY71 and GLY81; see Table S7. Structure is additionally stabilized by one water bridges binding the ligand with PHE149.

Thus, the binding pattern of the native ligand, SAM, shows more variation in residues among different CoVs than sinefungin.

### 3.2. Step II: The Pre-Selection of Ligands

Known inhibitors, including sinefungin, tubercidin, toyocamycin, sangivamycin, 5-iodotubercidin, adenosine and S-adenosylhomocysteine were optimized at the M062X/6-31G(d,p) level of the theory and prepared for docking. 

A set of 967 pre-selected ligands, structurally resembling SAM, the so-called molecular chameleons, was established based on a 3D conformational fingerprint template (SMILES) with a 90% Tanimoto coefficient. Their structures were subjected to verification and optimization at the M062X/6-31G(d,p) level of the theory. In principle, a straightforward geometry optimization using classical molecular mechanics would be sufficient. However, the pre-selected ligands possess numerous degrees of freedom, and the selection of an equilibrium conformation while disregarding electronic effects can have significant and far-reaching consequences [53,54].

### 3.3. Step III: The Pre-Selected Ligands Docking and Screening

Subsequently, the pre-selected ligands were docked in the Nsp16 protein pocket. The 6W4H structure was used for this bulk task. The atomic hybridization and protonation state of the protein was checked and corrected prior to docking. The total time for the single docking attempt of the entire set of ligands about 1000 ligands was several hours. The same procedure was used to establish protein–ligand models for all ligands. 

The binding affinity prediction was used to rank ligands considering the top energy poses of each compound. For each pre-selected ligand, the structure–binding affinity index, SBAI, was calculated according to Equation (3), with the 3D pharmacophore similarity as s. 

**Figure 7:**
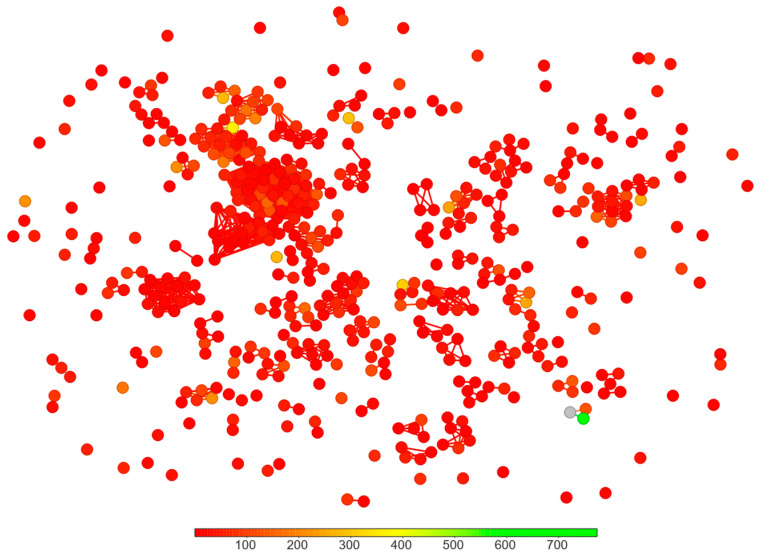
The chart showing pre-selected and rejected ligands (low absolute SBAI—red/high absolute SBAI—green).

The chart showing rejected/pre-selected ligands is shown in Figure 7. The color spectrum ranges from red (low absolute SBAI) to green (high absolute SBAI).

During the screening, it was found that all 2′-deoxy derivatives have weaker binding affinity ad thus were rejected. A further study was conducted on 8 already known inhibitors and 10 candidate ligands selected based on high absolute SBAI vales. The chemical names of the selected ligands and Tanimoto coefficient (used exclusively in the pre-selection task, Step II) as well as 3D pharmacophore similarity coefficients (used for the calculation of the similarity parameter s in SBAI, Equation (3) and subsequent candidate selection) are summarized in Table 6. 

As follows from Table 6, the Tanimoto and 3D pharmacophore coefficients are poorly correlated (the Pearson’s correlation coefficients are low and do not exceed 0.538). Thus the manner in which similarity in Equation (3) is calculated matters significantly. The use of the 3D pharmacophore, which is defined as an ensemble of structural features that are crucial to attach or bind to an active site of an enzyme or molecule [81], helps to incorporate not only the ligand’s shape or conformation, but also its entire set of features (e.g., hydrogen bond donors, hydrogen bond acceptors, charged groups and hydrophobic regions).

### 3.4. Step IV: The Docking of the Candidate Ligands and Known Inhibitors

Six target structures were used in the further MD study: three with the native SAM ligand (6WH4, 5YN6 and 3R24) and three with the inhibitor sinefungin (6WKQ, 5YNB and 2XYR). The RMSD of the pose of the native ligand from its conformation in the parent structure did not exceed 0.3 Å. The docking procedure of the 17 candidate ligands and known inhibitors was conducted in accordance with the established protocol.

**Figure 8:**
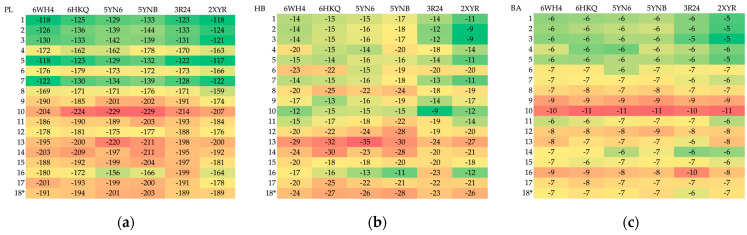
A comparison of the heatmaps visualizing (**a**) protein–ligand energy, PL, (**b**) hydrogen bond term, HB and (**c**) binding affinity, BA, for 102 complexes under investigation (* reference ligand). (Red represents favorable values, while green indicates unfavorable values.).

**Figure 9:**
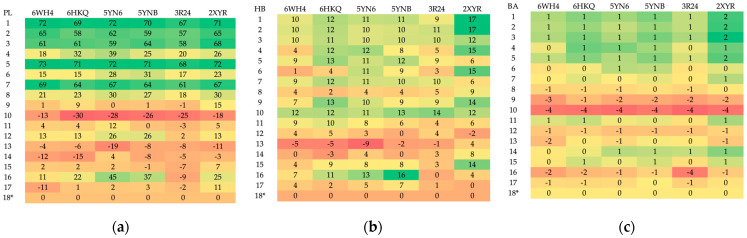
Different heatmaps visualizing the relative (**a**) protein–ligand, (**b**) hydrogen bonding (**c**) binding affinity among the set of 102 structures (* reference ligand). (Red indicates an increase and green a decrease in value).

A total of 102 complexes obtained during MD were subjected to analysis. The docking results are summarized in Table S8 and visualized as heatmaps in Figure 8 and Figure 9.

The candidate ligands appear to be more promising than 8 already known inhibitors. They bind more strongly to the targets; see Figure 8 and Figure 9. In general, the protein–ligand binding energy depends by about 42% on the number of hydrogen bonds and 46% on the strength of hydrogen bonds, about 58% on the number of heavy atoms and nearly 95% on steric interactions. In the context of the pocket construction and binding mode analysis, both the electrostatic and hydrophobic interactions are crucial factors.

The degree of similarity between the ligands measured by Tanimoto similarity varies from 63.58 to 98% and reaches 98% in ligand 8 (SAH). The degree of similarity between the ligands measured by the 3D pharmacophore similarity varies from 80 to 98% and reaches 98% in ligand 11. However, the binding affinity for this ligand is not the highest. The MD results do not seem to correlate directly with the classical Tanimoto similarity; the 3D pharmacophore similarity seems a better choice. A rough analysis of the data listed in Table S8 and shown in Figure 9 suggests that ligand 10 should represent the optimal choice.

### 3.5. Step V: In-Depth Investigation of Protein–Ligand Binding

#### 3.5.1. Classical In-Depth Analysis

**Figure 10:**
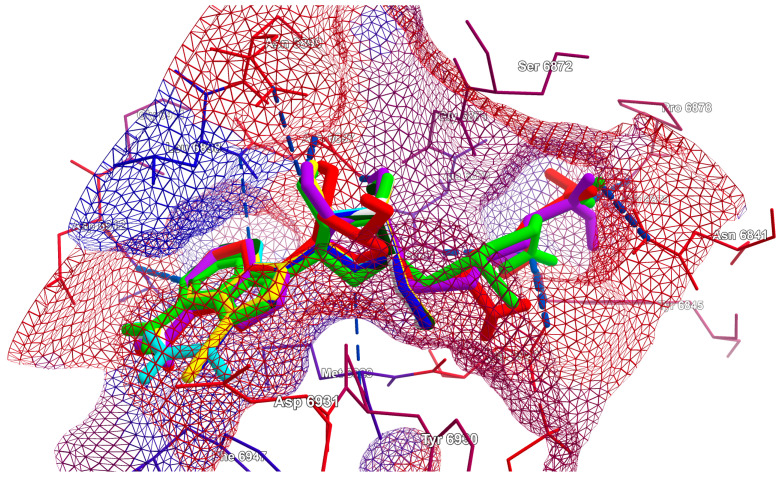
The best poses of the known inhibitors: 1 (dark green), 2 (yellow), 3 (cyan), 4 (green), 5 (white), 6 (red), 7 (blue) and 8 (violet); target from 6WH4. The hydrogen bonds between the ligand and the Nsp16 residues are shown with a blue dashed line. The residues are colored according to their hydrophobicity. The colors of the hydrophobic surface reflect the hydrophobicity of the residues.

**Figure 11:**
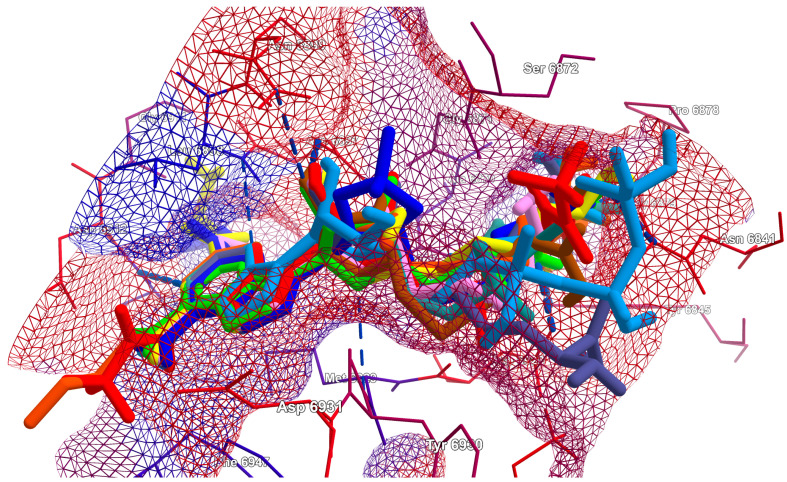
The best poses of the candidate ligands: 9 (brown), 10 (red), 11 (dark cyan), 12 (light pink), 13 (light blue), 14 (dark violet), 15 (orange), 16 (navy) and 17 (yellow). The native ligand, SAM, is shown in light green; target from 6WH4. The residues are colored according to their hydrophobicity. The colors of the hydrophobic surface reflect the hydrophobicity of the residues.

An in-depth investigation of the MD results reveals more details concerning the entire binding mode. In addition to the aforementioned docking scores, it is important to ensure that the necessary packing is carried out in the protein pocket. The best poses of the known inhibitors and candidate ligands are illustrated in Figure 10 and Figure 11, respectively.

The hydrogen bonds between the ligand and the Nsp16 residues are shown with a blue dashed line. The colors of the lines forming the hydrophobic surface grid reflect the hydrophobicity of the individual residues. The residues are colored according to their hydrophobicity. It is evident that the polarity of the pocket changes from hydrophilic (adenine moiety) to hydrophobic (glycone and methionine moieties). Substituents play a pivotal role in both the filling of the pocket and the orientation of the ligand within the pocket.

The highly polar substituent -CF_3_ of ligands 9 and 10 is responsible for effectively locking the ligand inside the pocket. However, the methionine moiety in ligands 10 and 9 is oriented in a mirrored manner; see Figure 11. In general, the candidate ligands are observed to fill the pocket to a greater extent than the known inhibitors. The assessment of the electrostatic match between ligands and binding pockets, in conjunction with the investigation of hydrophobicity, provides valuable insights into the underlying mechanisms of ligand binding and the potential for improving binding affinity; see Figure 9 and Figure 10. As a consequence of the uneven distribution of electrostatic and hydrophobic charges, the protein pocket is dipolar. The most optimal orientation for the ligand in the pocket is one that provides the greatest benefit, in terms of the ligand’s ability to interact with the protein. The most promising Nsp16 ligands are oriented in a manner that allows the adenine and methionine residues to be immersed in negative/hydrophobic and positive/hydrophilic potentials, respectively.

**Figure 12:**
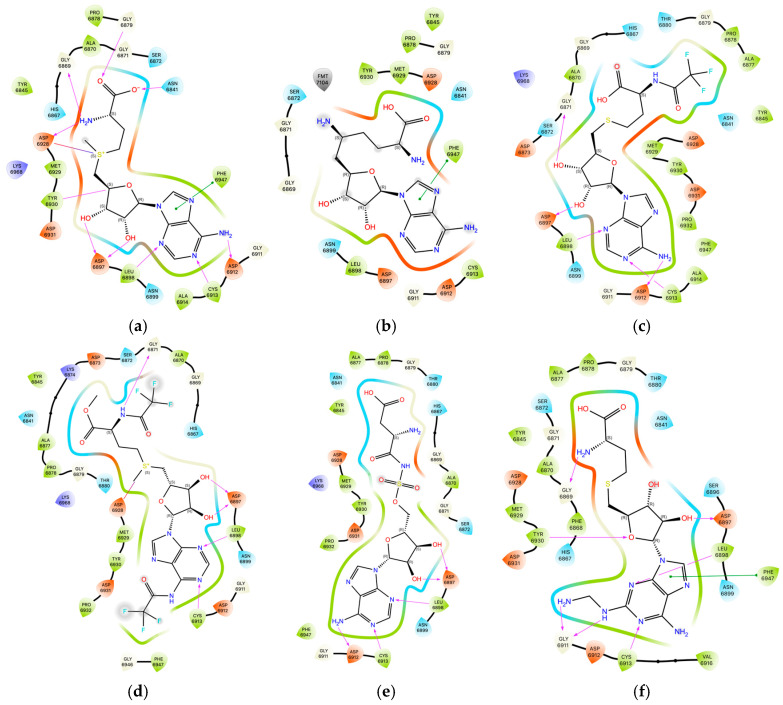
A comparison of the components of the binding pattern (hydrophobic interactions and hydrogen bonds) (**a**) SAM (**b**) sinefungin, (**c**) ligand 9 (**d**) ligand 10 (**e**) ligand 11 and (**f**) ligand 17. (The best poses are shown in Figure 9 and Figure 10.).

The number of the available hydrogen bond donors, acceptors and interactions involved in the ligand–protein binding (hydrogen bonds and hydrophobic) were quantified for each ligand (see Table 7) and visualized; see Figure 12. The analysis was carried out using [82,83].

The number of interactions differs significantly depending on the ligand type, with weekly fluctuations observed in the case of the viral Nsp16 protein type. For ligand 10, 8/9/10 hydrogen bonds and 11/11/10 hydrophobic interactions are formed in 6W4H/5YN6/3R24 targets, respectively. (The number of residues involved in hydrophobic interactions remains constant). The ligand with the greatest difference between the number of donors and acceptors exhibited the highest binding affinity. It is somewhat surprising that ligand 13, which has a considerable number of donor and acceptor groups and forms 12 hydrogen bonds with residues, binds relatively weekly. It seems that the optimal number of hydrogen bond donors and acceptors in ligands should not exceed 8 and 12, respectively.

**Figure 13:**
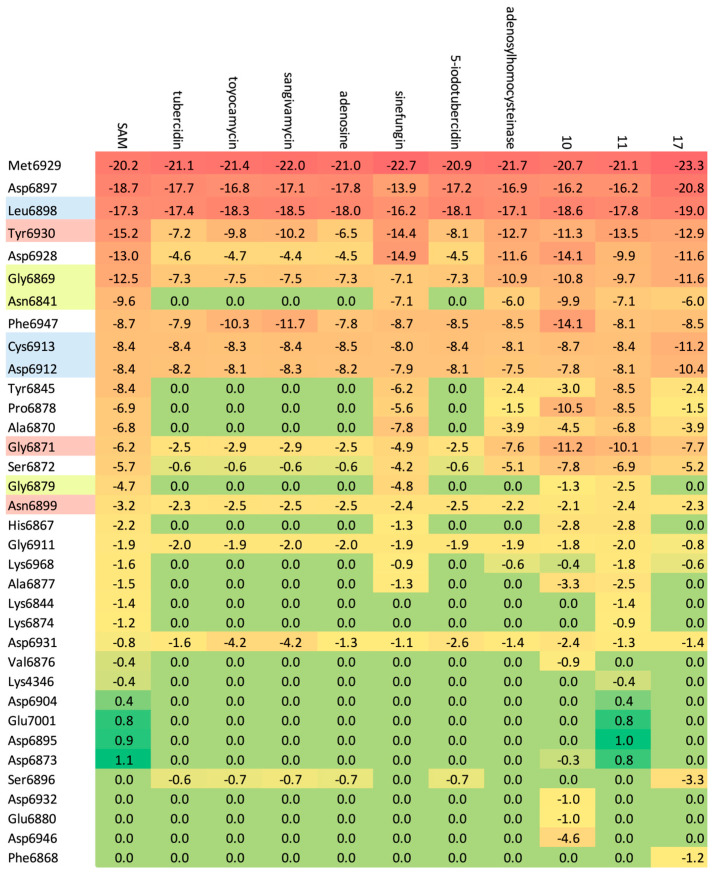
Heatmap showing the differences in the binding patterns between the different ligands; 6WH4 target. Negative contributions are marked in red; positive contributions are marked in green. The names of the key residues that bind the protein to the ligand via hydrogen bonds are marked with a color (adenine, methionine and glycone moieties in blue, yellow and pink, respectively).

By comparing the binding patterns for the native and candidate ligands, a particular effect of the resemblance of ligands 10 and 11 to SAM can be observed; see Figure 13.

The average distance between the binding modes is the smallest for ligand 11 (of 8 kcal/mol) and the highest for adenosine (of 72 kcal/mol). RMSD_BM, Equation (2), ranges from 30 kcal/mol for ligand 11 to 37 kcal/mol for sinefungin to 86 kcal/mol for adenosine. For the most promising ligands, i.e., 10 and 17, RMSD_BM is about 60 kcal/mol. The ligand change results in the largest differences at residues TYS6930, ASP6828, GLY6869, ASN6841, TYR 68590, PRO6878, ALA6970, GLY6871, SER 6872 and GLY 6879; see Figure 11. The most effective compounds are those that bind with the greatest strength to the residues, namely GLY6871, PHE6947, TYR6930 and ASP6928, which suggests that the glycone moiety of the ligand plays a key role. The low effectiveness of known inhibitors compared to candidate ligands also results from the weakened binding of methionine moiety (or its replacements) to protein residues GLY6869 and to a lesser extent ASN6841 and GLY6869; see Figure 13.

#### 3.5.2. Rapid Global Analysis

**Figure 14:**
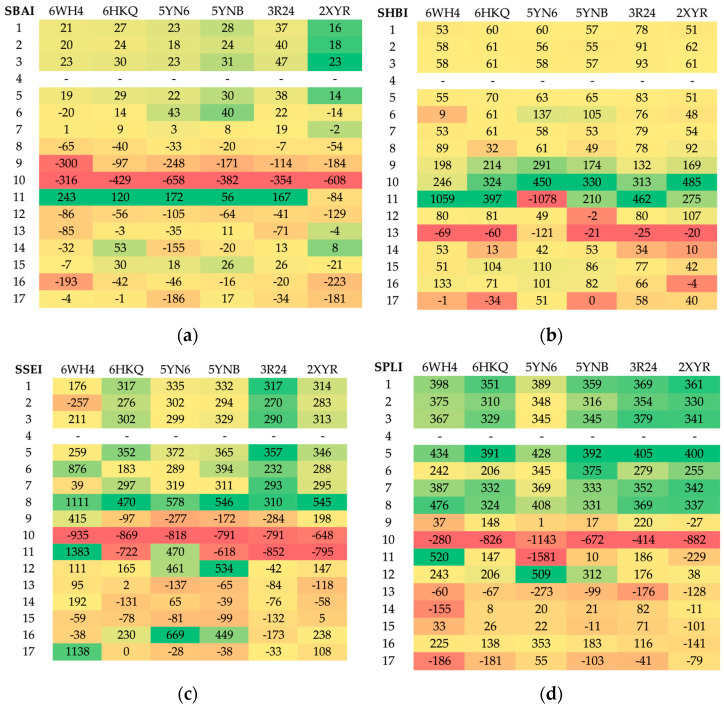
The heatmaps of SBS indices (**a**) structure–binding affinity index (SBAI), (**b**) structure–hydrogen bond index (SHBI), (**c**) structure–steric effect index (SSEI) and (**d**) structure–protein–ligand index (SPLI) for 102 complexes (17 ligands and six targets). (Red represents the negative values, while green indicates positive values.). Ligand 4 (SAM) is the reference.

As demonstrated above, the analysis of binding modes and finding most promising ligands is a complex process that necessitates the consideration of numerous factors. In order to facilitate such an analysis, we proposed the use of new indices. The four SBS indices SBAI, SHBI, SSEI and SPLI were calculated via Equations (3)–(6) and are shown in the heatmaps, which allow for the visual representation of the datasets in an easily understandable form; see Figure 14.

The greater the absolute value of the indices, the more promising the ligand. Negative values indicate an increase in protein–ligand binding strength, while positive values indicate its weakening relative to the reference ligand. Based on the SBAI and SPLI values, ligands 10 and 9 emerged as the most optimal candidates. The SBAI and SPLI values for them are highest in absolute value, which indicate strong binding to Nsp16 and high similarity to SAM. Both ligands contain at least one trifluoromethyl group, which has a strong electron-withdrawing nature, poor polarizability and a broad hydrophobic domain. This moiety increases the hydrophobicity (lipophilicity) of the ligand. Subsequent analysis based on SSEI revealed that ligands 10 and 11 are optimal from a steric point of view. If hydrogen bonds are of significant importance, then according to SHBI, ligands 13 and 17 will be a superior choice. However, ligand 13 contains the triphosphoric moiety, which is unfavorable. Although triphosphates represent the active form of the antiviral drugs, direct delivery of the triphosphates into cells is not a viable option, as these multi-charged molecules are unable to permeate lipophilic cell membranes.

The use of indices facilitates the assessment of selectivity; see Figure 13. The heatmaps indicate that ligands 11 and 16 should be the most selective. It can be postulated that the former should inhibit MERS-CoV and the latter SARS-CoV-2. Ligand 15 does not appear promising due to the butyl moiety, which is flexible but non-polar and hydrophobic.

On the other hand, ligands 16 and 17 were investigated to identify molecules that interact with methyltransferase and purify methyltransferase proteins (patent WO-2012016704-A1). Ligand 14 happened to be a coactivator-associated arginine methyltransferase 1 (CARM1) inhibitor (5ISE [84]). CARM1 is a protein arginine methyltransferase that asymmetrically dimethylates histone H3 and non-histone proteins to regulate gene transcription [85].

### 3.6. Step VI: Ligand Tuning

**Figure 15:**
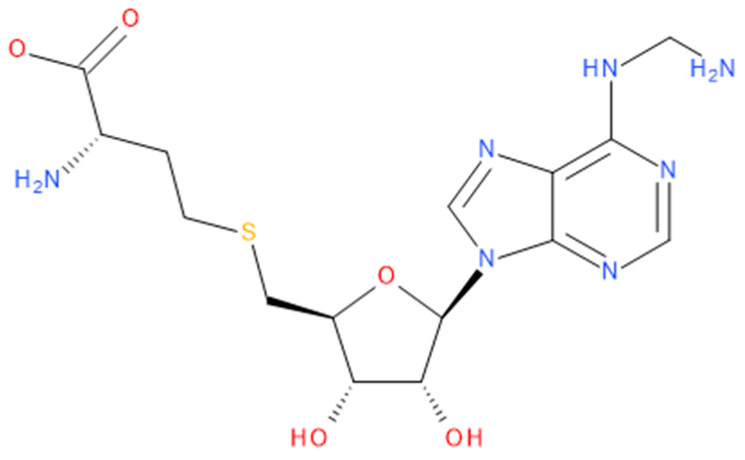
The structural formula of 2-Amino-4-[[5-[6-amino-2-(3-aminomethylamino)purin-9-yl]-3,4-dihydroxyoxolan-2-yl]methylsulfanyl]butanoic acid).

Based on abovementioned analysis, the modification of the amine group in the adenine moiety from -NH_2_CH_2_CH_2_CH_2_NH_2_ to -NH_2_CH_2_NH_2_ in ligand 17 was proposed; see Figure 15. To the best of our knowledge, this compound has never been synthesized.

**Figure 16:**
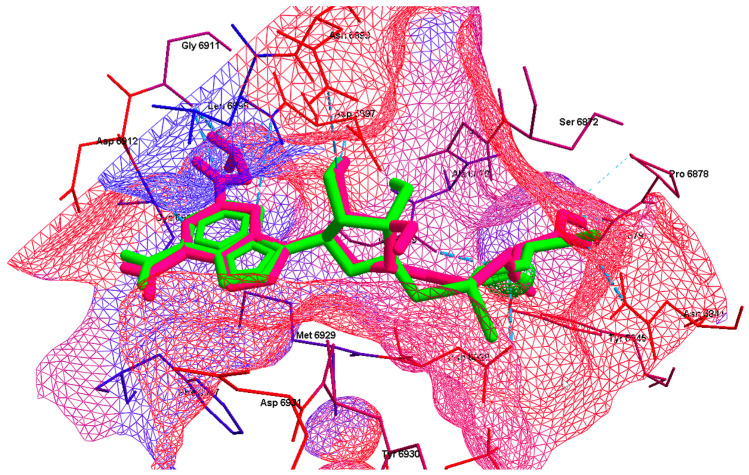
The best pose of the new ligand 2-Amino-4-[[5-[6-amino-2-(3-aminomethylamino)purin-9-yl]-3,4-dihydroxyoxolan-2-yl]methylsulfanyl]butanoic acid (magenta). The native ligand, SAM, is shown in light green; target—6WH4. The hydrogen bonds between the ligand and the Nsp16 residues are shown with a blue dashed line. The residues are colored according to their hydrophobicity. The colors of the hydrophobic surface reflect the hydrophobicity of the residues.

**Figure 17:**
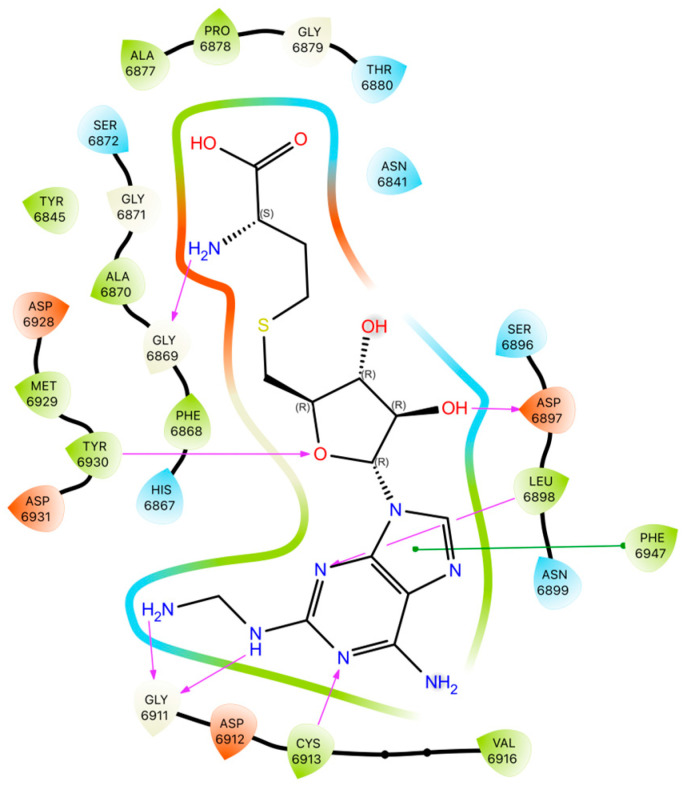
The components of the binding pattern: hydrophobic interactions and hydrogen bonds, (2~{S})-2-amino-4-[[(2~{S},3~{S},4~{R},5~{R})-5-[6-(aminomethylamino)purin-9-yl]-3,4-dihydroxy-tetrahydrofuran-2-yl]methylsulfanyl]butanoic acid—Nsp16 complex.

The (2~{S})-2-amino-4-[[(2~{S},3~{S},4~{R},5~{R})-5-[6-(aminomethylamino)purin-9-yl]-3,4-dihydroxy-tetrahydrofuran-2-yl]methylsulfanyl]butanoic acid ligand was docked in the pocket in 6WH4. The outcome was a notable change in all four indices: SBAI = −316, SHBI = −70, SPLI = −280 and SSEI = −402, which are negative and of a considerable magnitude. Indeed, the binding affinity of this modified ligand, 2~{S})-2-amino-4-[[(2~{S},3~{S},4~{R},5~{R})-5-[6-(aminomethylamino)purin-9-yl]-3,4-dihydroxy-tetrahydrofuran-2-yl]methylsulfanyl]butanoic acid to Nsp16 from SARS-CoV-2, is −9.88 kcal/mol, and the hydrogen bond and protein–ligand interactions are −22.46 and −203.70 kcal/mol, respectively. The aforementioned values are the most proximate to those for ligand 10. The 3D pharmacophore similarity of the new ligand to SAM is 94%. Despite the high similarity of the binding mode, the RMSD_BM distance is relatively high at 141 kcal/mol, which is caused by the stronger binding to GLY6911, ASP6912, TYR6930 and Lys6968 and ASP6932; see Figure 12, Figure 16 and Figure 17. The binding of the new ligand to Nsp16 from SARS-CoV and MERS-CoV is weaker, at −6.35 and −6.26 kcal/mol.

The best pose of the 2~{S})-2-amino-4-[[(2~{S},3~{S},4~{R},5~{R})-5-[6-(aminomethylamino)purin-9-yl]-3,4-dihydroxy-tetrahydrofuran-2-yl]methylsulfanyl]butanoic acid is shown in Figure 16.

2~{S})-2-amino-4-[[(2~{S},3~{S},4~{R},5~{R})-5-[6-(aminomethylamino)purin-9-yl]-3,4-dihydroxy-tetrahydrofuran-2-yl]methylsulfanyl]butanoic acid forms hydrogen bonds with CYS6913, GLY6911 and SER6896 (complementary to the protein–ligand complex with SAM), binds to GLY6872 with a hydrogen bond instead of a hydrophobic interaction and participates in hydrophobic interactions with ALA6877 instead of SER6872, but, unlike SAM, it does not bind to ASP6897. The ligand is oriented with the adenine and methionine residues to be immersed in negative/hydrophobic and positive/hydrophilic potentials, respectively. Polar glycone moiety forms a kind of bridge between both parts of the molecule.

### 3.7. Step VII: Drug-Likeness Validation—ADMET Evaluation of the Ligands

**Figure 18:**
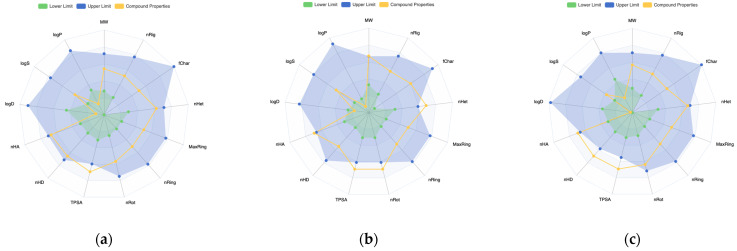
The radar view of physicochemical properties (**a**) SAM, (**b**) ligand 10, (**c**) new ligand (2~{S})-2-amino-4-[[(2~{S},3~{S},4~{R},5~{R})-5-[6-(aminomethylamino)purin-9-yl]-3,4-dihydroxy-tetrahydrofuran-2-yl]methylsulfanyl]butanoic acid; ADMET 3.0.

The pharmacokinetic parameters that a given inhibitor should exhibit depends on the specific target requirements. The druggability of the ligands can be verified based on ADMET analysis. In general, the ligand candidates have better physicochemical than already known inhibitors. The parameters describing the physicochemical profile and pharmacokinetics behavior as well as toxicity of the candidate ligands estimated using different ADMET [61,62] protocols are listed in Tables S9 and S10 and shown in Figure 18.

The flexibility of the ligand candidates is higher than that of known inhibitors. Moreover, for ligands 10 and 17, it is the highest, so flexibility seems to be a key factor. The ligands 9 and 10 have trifluoromethyl groups, which should alter their solubility. However, their profile is not optimal. ADMET parameters suggests that compound 17 has a better pharmacokinetic profile; higher QED; lower carcinogenicity; low AMES, H-HT and DILI; optimal solubility; an octanol–water partition coefficient; synthesis accessibility; SAS; PAMPA; and CaCo-2. Moreover, the so-called “golden triangle rule” is not violated. The lack of halogens is not a challenge but rather a favorable circumstance from the point of view of formation of hydrogen bonds and toxicity. The physicochemical, pharmacokinetic and toxicity profiles of the new ligand (2-Amino-4-[[5-[6-amino-2-(3-aminomethylamino)purin-9-yl]-3,4-dihydroxyoxolan-2-yl]methylsulfanyl]butanoic acid) are not worse than ligand 17. It is important to note that there is no definitive set of ideal pharmacokinetic parameters that a given inhibitor should exhibit, as these depend on the specific requirements of the target in question. ADMET, which can be regarded as a form of support for the design process, but not as a criterion for selection, suggests that the pharmacological profile exhibited by prospective ligands is comparable to that demonstrated by known inhibitors.

### 3.8. Step VIII: Stability Evaluation—Molecular Dynamic Simulations

**Figure 19:**
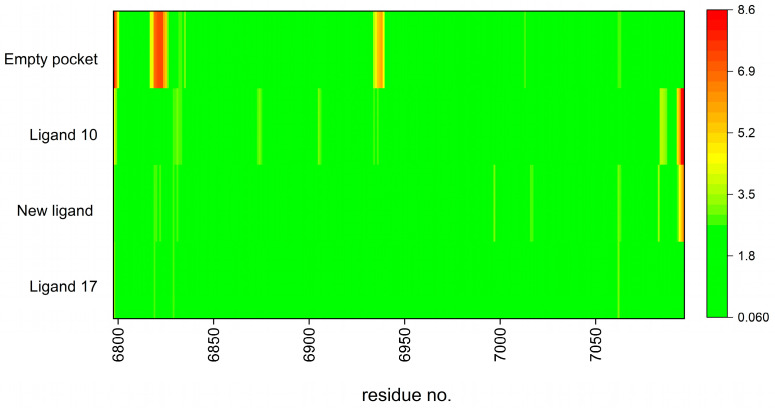
Root means square fluctuation (RMSF) graph representing MDS of the individual residues. The color scale bar is displayed in the image; the most flexible residues are shown in red.

The weak point of classical molecular docking is the limited flexibility of the residues. We therefore applied two different docking techniques: flexible ligands and flexible residues, the latter being used to the extent possible. Nevertheless, further investigation is necessary to address this issue in a slightly more comprehensive way. Molecular dynamics simulations (MS) were performed using a coarse-grained approach. First, the flexibility of the protein residues was checked in the absence of the ligands. In the next step the entire protein–ligand complex was examined. The root mean square fluctuation (RMSF) of a structure which represent the root mean square deviation of atomic positions from their mean positions over time (RMSD) and provide information about the flexibility and dynamics of the complexes; see Figure 19. It is worth noting that the binding site is relatively stable even when the pocket is empty. The high flexibility was observed only nearly the three residues: GLU6821, LYS6822 and THR6938 (RMSF is equal 7.30, 7.45 and 6.03 Å, respectively).

The RMSF fluctuation plot, depicted in Figure 19, illustrates the residue-wise fluctuations observed throughout the simulation.

**Figure 20:**
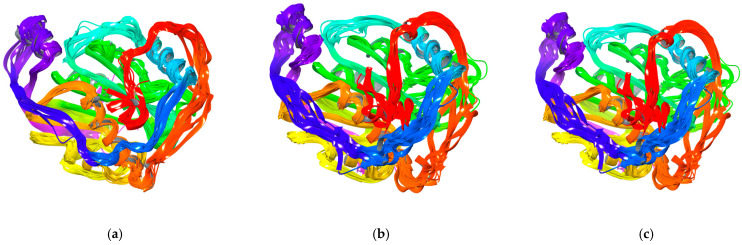
Multimodel (several models superimposed) visualization of the fluctuations of the Nsp16 complexed with (**a**) ligand 10, (**b**) ligand 17, (**c**) (2-Amino-4-[[5-[6-amino-2-(3-aminomethylamino)purin-9-yl]-3,4-dihydroxyoxolan-2-yl]methylsulfanyl]butanoic acid); residue position color scheme was used.

For the protein–ligand complexes, the RMSF value does not exceed 3.5 Å. (The terminal residues in ligands 10 and the newly designed ligand exhibit a scattering from this trend). The radius of gyration changed relatively little, by no more than 4%. The multimodel (several main models superimposed) visualization of the fluctuations is shown in Figure 20.

The differences in binding modes are within the range produced by different docking techniques and do not affect the final conclusions. The ligands presence stiffens the whole complexes.

## 4. Conclusions

The aim of our study was to propose and validate an effective screening protocol dedicated to identify potential drug candidates that may act as Nsp16 inhibitors and reveal the presence of the virus to the immune defense system. To date, only a limited number of such inhibitors have been identified.

Our innovative approach includes eight well-defined steps, and the entire process, called the chameleon strategy, is shown in the flowchart. The core this methodology is the use of novel SBSI indices. Structure–binding affinity index (SBAI), structure–hydrogen bonding index (SHBI), structure–steric effect index (SSEI) and structure–protein–ligand index (SPLI) are proposed in this paper to screen SAM analogues to reveal the extent to which binding affinity is gained or lost in response to the relatively small changes in the ligand structure.

The proposed approach has proven to be effective in the search for suitable SAM analogues and Nsp16 inhibitors. Combined data mining using the SAM/SAH structure as a template and Tanimoto similarity as an acceptance/rejection criterion helped to identify 967 candidate ligands with relatively high structural 3D similarity to SAM, the so-called molecular chameleons. Then, molecular docking supported by the new SBAI index reduced the chemical space in the search for ligands that capable of binding to the target protein and revealed 10 candidate ligands. A dedicated approach designed for in-depth analysis of the protein–ligand binding modes based on the mathematical metrics and heatmaps helped analyze protein–ligand binding modes. But in identifying three highly promising ligands and designing the new one, 2~{S})-2-amino-4-[[(2~{S},3~{S},4~{R},5~{R})-5-[6-(aminomethylamino)purin-9-yl]-3,4-dihydroxy-tetrahydrofuran-2-yl]methylsulfanyl]butanoic acid, four novel SBSI indices were proved to be the most efficient.

As demonstrated above, this methodology can be successfully applied not only to screen and tune ligands, but also to determine the effectiveness of the ligand in response to changes in the target viral entity, which is particularly useful for assessing drug effectiveness in the case of alterations in viral proteins.

The developed strategy is so general that it can effectively support screening tests and set new directions for improving inhibitors, not only antiviral ones. In conclusion, the chameleon strategy has the capacity to introduce a novel universal paradigm to the field of RNA antiviral design and drug design in general.

## Figures and Tables

**Table 1:** The classification, host cell receptors and genotype resemblance of SARS-CoV, MERS-CoV and SARS-CoV-2.

Virus	First Appearance	Genus	Receptor	Dominant Host Receptor	Co-Receptors	Length of Nucleotides [Kilobases]	Genotype Resemblance to SARS-CoV-2
SARS-CoV-2	7/12/2019 Wuhan, China	Clade I, lineage B	cellular serine protease TMPRSS2; endosomal cysteine proteases cathepsin B/L	angiotensin-converting enzyme 2 (ACE-2)	CD147-SP	29.9	100%
SARS-CoV	16/11/2002 Foshan, China	Clade I, lineage B	cellular serine protease TMPRSS2; endosomal cysteine proteases cathepsin B/L	angiotensin-converting enzyme 2 (ACE-2)	DC-SIGN (CD209) L-SIGN (CD209L)	29.75	79%
MERS-CoV	4/04/2012 Zarqa, Jordan	Clade I, lineage C	cellular serine protease TMPRSS2; endosomal cysteine proteases cathepsin B/L	dipeptidyl peptidase 4 (DPP4, CD26)	-	30.11	50%

**Table 2:** A comparison of the key roles of the most important targets.

Function	S Protein	E Protein	M Protein	N Protein	Nsp Proteins
binding to cell receptor	+	−	−	−	−
mediate cell fusion	+	−	−	−	−
virus assembly	−	+	+	+	+
morphogenesis	−	+		−	−
virus replication	−	−	−	+	+
immune response	−	−	−	+	+
modulation	−	−	−	+	+

The plus sign (+) indicates that the function is active and the minus sign (−) indicates that it is inactive.

**Table 3:** The numbers of the experimental structures of SARS-CoV-2, SARS-CoV-1 and MERS-CoV.

	Experimental Structural Data					
	Total	X-ray	Electron Microscopy	Solution NMR	Neutron Diffraction	Solid State NMR
SARS-CoV-2	4156	2805	1331	16	7	4
SARS-CoV						
MERS-CoV	323	242	78	3	-	-

**Table 4:** A comparison of the chain A resemblance between the pairs of non-structural Nsp16 proteins.

	(6W4H, 6WKQ)	(5YN6, 5YNB)		(3R24, 2XYR)	
RMSD [Å]	0.798	0.703		0.6363	
Different residues	ALA6798 SER6799	LYS31 GLU32 SER33 ILE34	GLY141 VAL294 LEU295 VAL296	TYR136 LYS137 HIS138 VAL139	ASP293 ILE294

**Table 5:** Anatomy of protein pockets.

	SARS-CoV-2		MERS-CoV		SARS-CoV	
	6W4H	6WKQ	5YN6	5YNB	3R24	2XYR
Acceptors	24	27	22	26	27	34
Donors	17	22	17	18	21	25
Depth [Å]	21.67	21.82	22.78	22.88	20.92	22.87
Surface [Å^2^]	1016.91	1218.48	1052.29	1055.14	1160.49	1410.73
Volume [Å^3^]	707.58	762.88	680.96	701.95	731.65	918.02
Surface–Volume Ratio	1.44	1.60	1.55	1.50	1.59	1.54
Hydrophobicity	0.56	0.59	0.64	0.64	0.61	0.64

**Table 6:** The list of inhibitors and candidate ligands and their similarity to S-adenosyl-L-methionine (SAM).

No.	Chemical Name	Chemical Structure	Tanimoto Similarity to SAM [%]	3D Pharmacophore Similarity
1	**Tubercidin** (2R,3R,4S,5R)-2-(4-aminopyrrolo[2,3-d]pyrimidin-7-yl)-5-(hydroxymethyl)oxolane-3,4-diol	** 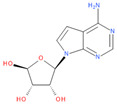 **	65.45	0.80
2	**Toyocamycin** 4-amino-7-[(2R,3R,4S,5R)-3,4-dihydroxy-5-(hydroxymethyl)oxolan-2-yl]pyrrolo[2,3-d]pyrimidine-5-carbonitrile	** 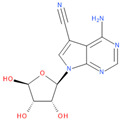 **	63.91	0.81
3	**Sangivamycin** 4-amino-7-[(2R,3R,4S,5R)-3,4-dihydroxy-5-(hydroxymethyl)oxolan-2-yl]pyrrolo[2,3-d]pyrimidine-5-carboxamide	** 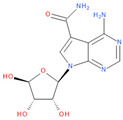 **	63.58	0.81
4	**S-adenosyl-L-methionine, SAM** (2S)-2-amino-4-[[(2S,3S,4R,5R)-5-(6-aminopurin-9-yl)-3,4-dihydroxyoxolan-2-yl]methyl-methylsulfonio]butanoate	** 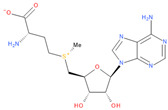 **	100.00	1.00
5	**Adenosine** (2R,3R,4S,5R)-2-(6-aminopurin-9-yl)-5-(hydroxymethyl)oxolane-3,4-diol	** 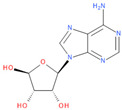 **	86.39	0.82
6	**Sinefungin** (2S,5S)-2,5-diamino-6-[(2R,3S,4R,5R)-5-(6-aminopurin-9-yl)-3,4-dihydroxyoxolan-2-yl]hexanoic acid	** 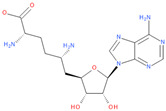 **	83.81	0.93
7	**5-Iodotubercidin** (2R,3R,4S,5R)-2-(4-amino-5-iodopyrrolo[2,3-d]pyrimidin-7-yl)-5-(hydroxymethyl)oxolane-3,4-diol	** 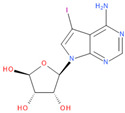 **	64.44	0.81
8	**S-adenosylhomocysteine, SAH** (2S)-2-amino-4-[[(2S,3S,4R,5R)-5-(6-aminopurin-9-yl)-3,4-dihydroxyoxolan-2-yl]methylsulfanyl]butanoic acid	** 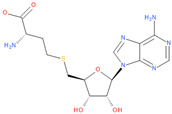 **	98.23	0.93
9	(2S)-4-[[(2S,3S,4R,5R)-5-(6-aminopurin-9-yl)-3,4-dihydroxyoxolan-2-yl]methylsulfanyl]-2-[(2,2,2-trifluoroacetyl)amino]butanoic acid	** 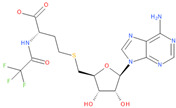 **	93.22	0.94
10	[(2S,3S,4R,5R)-3,4-dihydroxy-5-[6-[(2,2,2-trifluoroacetyl)amino]purin-9-yl]oxolan-2-yl]methyl-[4-methoxy-4-oxo-3-[(2,2,2-trifluoroacetyl)amino]butyl]-methylsulfanium	** 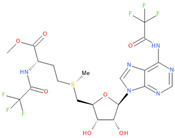 **	85.77	0.96
11	**5′-O-[N-(L-Aspartyl)sulfamoyl]adenosine** (3S,4Z)-3-amino-4-[[(2R,3S,4R,5R)-5-(6-aminopurin-9-yl)-3,4-dihydroxyoxolan-2-yl]methoxysulfonylimino]-4-oxidobutanoate	** 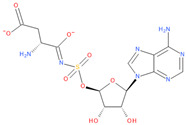 **	78.01	0.98
12	2-amino-4-(((2S,3S,4R,5R)-5-(6-amino-2-methoxy-9H-purin-9-yl)-3,4-dihydroxy-tetrahydrofuran-2-yl)methylthio)butanoic acid	** 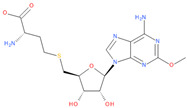 **	89.37	0.94
13	2-amino-4-[(1S)-1-[(2S,3S,4R)-5-(6-aminopurin-9-yl)-3,4-dihydroxyoxolan-2-yl]-2-[[hydroxy-[hydroxy(phosphonooxy)phosphoryl]oxyphosphoryl]amino]ethyl]sulfanylbutanoic acid	** 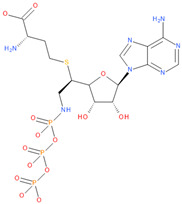 **	80.66	0.91
14	**6D2** 5′-{[(3s)-3-Amino-3-Carboxypropyl](3-Carbamimidamidopropyl)amino}-5′-Deoxyadenosine	** 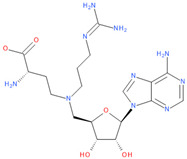 **	89.15	0.87
15	2-amino-4-[[5-[6-(butylamino)purin-9-yl]-3,4-dihydroxyoxolan-2-yl]methylsulfanyl]butanoic acid	** 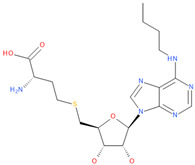 **	91.82	0.92
16	(2S)-4-[[(3aR,4R,6S,6aR)-4-[6-amino-2-(3-aminopropylamino)purin-9-yl]-2,2-dimethyl-3a,4,6,6a-tetrahydrofuro[3,4-d][1,3]dioxol-6-yl]methylsulfanyl]-2-aminobutanoic acid	** 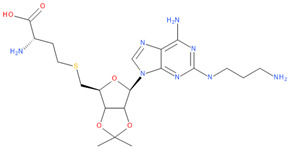 **	81.14	0.84
17	2-Amino-4-[[5-[6-amino-2-(3-aminopropylamino)purin-9-yl]-3,4-dihydroxyoxolan-2-yl]methylsulfanyl]butanoic acid	** 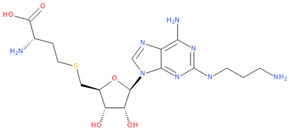 **	82.26	0.93

**Table 7:** The numbers of the donors, acceptors and interactions participating in the binding pattern in studied ligands.

No.	Donors	Acceptors	Hydrogen Bonds in Total	Adenine Moiety	Glycone Moiety	Methionine Moiety (or Its Replacement)	Hydrophobic Interactions in Total
1	4	7	4	1	2	1	8
2	4	8	5	1	4	-	7
3	5	8	5	1	4	-	8
4	4	10	7	2	2	4	8
5	4	8	5	2	3	-	7
6	6	11	8	2	2	5	7
7	4	7	4	1	3	-	7
8	5	11	8	2	2	4	9
9	5	14	8	2	2	4	10
10	4	16	8	3	3	2	11
11	4	15	8	2	3	3	10
12	5	12	8	2	2	4	7
13	10	21	12	2	2	8	7
14	7	12	9	2	2	5	11
15	5	11	7	2	1	4	11
16	5	13	5	3	-	2	14
17	7	13	11	3	1	7	12
New	8	12	11	4	1	6	7

## Data Availability

All data are included in the paper.

## Supplementary Materials

The following supporting information can be downloaded at: https://www.mdpi.com/article/10.3390/v16071073/s1, Table S1: The binding mode of the native ligand S-adenosylmethionine (SAM), SARS-CoV 2 (co-crystal 6W4H [38]); Table S2: The binding mode of the native ligand S-adenosylmethionine (SAM), SARS-CoV-2 (co-crystal from 6XKM [61]); Table S3: The binding mode of the native ligand Sinefungin, SARS-CoV-2 (co-crystal from 6WKQ [38]); Table S4: The binding mode of the native ligand S-adenosylmethionine (SAM), MERS-CoV (co-crystal from 5YN6 [59]); Table S5: The binding mode of sinefungin, MERS-CoV (co-crystal from 5YNB [59]); Table S6: The binding mode of the native ligand S-adenosylmethionine (SAM), SARS-CoV (co-crystal from 3R24 [60]); Table S7: The binding mode of the native ligand sinefungin, SARS-CoV (co-crystal from 2XYR [60]); Table S8: The docking results: PL—protein -ligand, HB—hydrogen bonds, BA—binding affinity and RMSD expressed in kcal/mol and Å, respectively; Table S9: The physico-chemical parameters describing the studied ligands; Table S10: The pharmacokinetic and toxicity profiles of the studied ligands.
