# Supplementary material for: The Chameleon Strategy—A Recipe for Effective Ligand Screening for Viral Targets Based on Four Novel Structure–Binding Strength Indices
Source: Viruses. 2024 Jul 3;16(7):1073. doi: 10.3390/v16071073 (PMC11281727; doi:10.3390/v16071073)
Supplement: Supplementary file 1 [file viruses-16-01073-s001.zip › viruses-3083273-supplementary.pdf]

viruses

MDPI

Suplementary

The Chameleon Strategy – A Recipe for Effective Ligand Screening for Viral Targets Based on Four Novel Structure-Binding Strength Indices

Magdalena Latosińska and Jolanta Natalia Latosińska *

Table S1. The binding mode of the native ligand S-adenosylmethionine (SAM), Sars-CoV 2 (co-crystal 6W4H [38]).

SAM moiety	Residue	Distance H-A [Å]	Distance D-A [Å]	D-A Angle [°]	Donor Atom	Acceptor Atom
methionine	ASN6841	1.82	2.74	153.20	391 [Nam]	3533 [O from CO2]
methionine	GLY6869	1.99	2.89	144.70	3529 [N3]	619 [O2]
methionine	GLY6879	1.96	2.86	149.92	675 [Nam]	3532 [O from CO2]
glycone	GLY6871	2.63	3.43	139.55	3542 [O3]	628 [O2]
glycone	ASN6899	2.95	3.30	102.53	852 [Nam]	3544 [O3]
glycone	TYR6930	2.50	3.38	147.68	1115 [Nam]	3540 [O3]
adenine	LEU6898	2.52	3.26	132.42	837 [Nam]	3554 [Nar]
adenine	ASP6912	3.05	3.91	152.39	972 [O3]	3552 [Nar]
adenine	ASP6912	2.64	3.59	160.42	3551 [Npl]	972 [O3]
adenine	CYP6913	2.35	3.03	122.89	976 [N3]	3552 [Nar]

Table S2. The binding mode of the native ligand S-adenosylmethionine (SAM), Sars-CoV 2 (co-crystal from 6XKM[62]).

SAM moiety	Residue	Distance H-A [Å]	Distance D-A [Å]	D-A Angle [°]	Donor Atom	Acceptor Atom
methionine	ASN43	1.93	2.65	127.46	335 [Nam]	3211 [O from CO2]
methionine	TYR47	2.31	3.04	134.89	368 [O3]	3208 [N3]
methionine	GLY71	2.05	2.89	137.57	3208 [N3]	563 [O2]
methionine	ASP130	1.73	2.65	168.14	995 [O3]	3208 [N3]
methionine	GLY81	2.24	3.15	153.03	619 [Nam]	3212 [O from CO2]
glycone	GLY73	2.72	3.49	137.17	3221 [O3]	572 [O2]
glycone	LEU100	3.31	4.04	131.94	762 [Nam]	3223 [O3]
glycone	ASN101	2.92	3.27	101.99	777 [Nam]	3223 [O3]
glycone	TYR132	2.59	3.52	157.82	1004 [Nam]	3219 [O3]
adenine	ASP114	2.56	3.47	153.71	3230 [Npl]	870 [O3]
adenine	CYS115	2.11	3.04	156.00	872 [Nam]	3231 [Nar]

Table S3. The binding mode of the native ligand sinefungin, Sars-CoV 2 (co-crystal from 6WKQ [38]).

SAM moiety	Residue	Distance H-A [Å]	Distance D-A [Å]	D-A Angle [°]	Donor Atom	Acceptor Atom
methionine	ASN6841	1.87	2.81	159.57	3690 [Nam]	6635 [O from CO2]
methionine	TYR6845	3.24	4.04	143.67	6636 [O.co2]	3723 [O3]
methionine	TYR6845	2.16	2.91	135.76	3723 [O3]	6632 [N3]
methionine	GLY6869	1.88	2.76	142.96	6632 [N3]	3918 [O2]
methionine	GLY6879	2.00	2.91	151.28	3974 [Nam]	6636 [O from CO2]
methionine	ASP6928	1.82	2.75	176.77	4358 [O3]	6632 [N3]
glycone	GLY6871	2.61	3.38	136.31	6645 [O3]	3927 [O2]
glycone	LEU6898	3.23	4.01	138.13	4117 [Nam]	6647 [O3]
glycone	TYR6930	2.41	2.81	156.41	4367 [Nam]	6643 [O3]
adenine	ASP6912	3.15	3.60	111.80	4233 [O3]	6655 [Nar]
adenine	ASP6912	2.10	3.03	156.30	6654 [Npl]	4233 [O3]
adenine	CYS6913	1.96	2.91	161.12	4235 [Nam]	6655 [Nar]

Table S4. The binding mode of the native ligand S-adenosylmethionine (SAM), MERS-CoV (co-crystal from 5YN6 [60]).

SAM moiety	Residue	Distance H-A [Å]	Distance D-A [Å]	D-A Angle [°]	Donor Atom	Acceptor Atom
methionine	ASN43	1.61	2.49	147.36	283 [Nam]	3043 [O from CO2]
methionine	TYR47	2.20	2.92	132.79	317 [O3]	3040 [N3]
methionine	TYR47	3.32	4.03	134.82	3044 [O.co2]	317 [O3]
methionine	GLY71	1.96	2.83	140.91	3040 [N3]	504 [O2]
methionine	GLY81	2.07	2.99	154.91	561 [Nam]	3044 [O from CO2]
methionine	ASP130	1.92	2.85	172.34	944 [O3]	3040 [N3]
glycone	GLY73	2.51	3.19	127.15	3053 [O3]	513 [O2]
glycone	ASN101	2.95	3.69	133.71	3055 [O3]	724 [O2]
glycone	TYR132	2.55	3.42	147.95	953 [Nam]	3051 [O3]
adenine	LEU100	2.48	3.29	138.74	710 [Nam]	3065 [Nar]
adenine	ASP114	2.31	3.24	156.49	3062 [Npl]	824 [O3]
adenine	CYS115	1.96	2.86	149.72	826 [Nam]	3063 [Nar]

Table S5. The binding mode of sinefungin, MERS-CoV (co-crystal from 5YNB [60]).

SAM moiety	Residue	Distance H-A [Å]	Distance D-A [Å]	Donor Angle [°]	Donor Atom	Acceptor Atom
methionine	ASN43	1.50	2.41	150.14	309 [Nam]	3134 [O from CO2]
methionine	TYR47	1.96	2.78	145.30	343 [O3]	3130 [N3]
methionine	GLY71	1.97	2.83	139.69	3130 [N3]	530 [O2]
methionine	GLY81	2.24	3.16	155.52	587 [Nam]	3133 [O from CO2]
methionine	ASP130	1.82	2.75	169.08	969 [O3]	3130 [N3]
glycone	GLY73	2.29	3.03	132.53	3143 [O3]	539 [O2]
glycone	TYR132	2.38	3.31	157.39	979 [Nam]	3141 [O3]
adenine	LEU100	2.48	3.28	138.73	736 [Nam]	3155 [Nar]
adenine	ASP114	2.36	3.27	154.46	3152 [Npl]	850 [O3]
adenine	ASP114	3.19	3.63	111.28	850 [O3]	3153 [Nar]
adenine	CYS115	1.95	2.89	158.58	852 [Nam]	3153 [Nar]

Table S6. The binding mode of the native ligand S-adenosylmethionine (SAM), Sars-CoV (co-crystal from 3R24 [61]).

SAM moiety	Residue	Distance H-A [Å]	Distance D-A [Å]	D-A Angle [°]	Donor Atom	Acceptor Atom
methionine	TYR47	2.33	3.03	131.63	359 [O3]	3208 [N3]
methionine	GLY71	1.90	2.79	144.13	3208 [N3]	554 [O2]
methionine	GLY81	1.97	2.85	148.84	610 [Nam]	3212 [O from CO2]
methionine	ASP130	1.79	2.72	170.82	986 [O3]	3208 [N3]
glycone	GLY73	2.66	3.48	143.01	3221 [O3]	563 [O2]
glycone	TYR132	2.44	3.34	151.47	995 [Nam]	3219 [O3]
adenine	LEU100	2.33	3.15	140.08	753 [Nam]	3233 [Nar]
adenine	ASP114	3.05	4.00	162.34	3230 [Npl]	861 [O3]
adenine	CYS115	2.00	2.89	148.59	863 [Nam]	3231 [Nar]

Table S7. The binding mode of the native ligand sinefungin, Sars-CoV (co-crystal from 2XYR [61]).

SAM moiety	Residue	Distance H-A [Å]	Distance D-A [Å]	D-A Angle [°]	Donor Atom	Acceptor Atom
methionine	ASN43	1.75	2.57	138.44	326 [Nam]	3130 [O from CO2]
methionine	GLY71	1.98	2.85	142.64	3126 [N3]	554 [O2]
methionine	GLY81	2.14	3.00	145.95	610 [Nam]	3129 [O from CO2]
glycone	GLY73	2.33	3.10	135.11	3139 [O3]	563 [O2]
glycone	ASN101	3.12	3.83	131.88	3141 [O3]	767 [O2]
glycone	TYR132	2.52	3.40	149.30	995 [Nam]	3137 [O3]
adenine	LEU100	2.57	3.34	134.43	753 [Nam]	3151 [Nar]
adenine	ASP114	2.59	3.50	153.85	3148 [Npl]	861 [O2]
adenine	CYS115	2.21	3.17	163.73	863 [Nam]	3149 [Nar]
water bridge	PHE149	3.10	3.32	134.41	1092[NAM]	3148[Npl]

Table S8. The docking results: PL- protein -ligand, HB – hydrogen bonds, BA – binding affinity and RMSD expressed in kcal/mol and Å, respectively.

No	6W4H			6WKQ			5YN6			5YNB			3R24			2XYR		
	PL	HB	BA	PL	HB	BA	PL	HB	BA	PL	HB	BA	PL	HB	BA	PL	HB	BA
1	-118.32	-14.22	-5.71	-125.17	-14.88	-5.79	-129.47	-15.40	-5.84	-133.11	-16.61	-5.98	-122.94	-13.68	-5.65	-117.55	-10.76	-5.33
2	-126.01	-13.85	-5.82	-135.70	-15.26	-5.98	-139.36	-16.44	-6.11	-143.84	-17.56	-6.23	-132.65	-12.24	-5.65	-124.17	-9.10	-5.30
3	-129.53	-14.22	-5.70	-133.08	-15.43	-5.73	-141.63	-16.42	-5.93	-139.40	-17.30	-5.94	-131.11	-12.49	-5.42	-120.64	-8.95	-5.02
4	-172.44	-20.03	-6.30	-161.94	-14.83	-5.62	-161.93	-14.02	-5.52	-177.64	-19.56	-6.25	-169.92	-17.78	-6.03	-162.94	-13.61	-5.59
5	-118.09	-14.72	-5.87	-123.30	-14.16	-5.80	-128.71	-15.87	-5.99	-131.99	-16.04	-6.01	-121.59	-14.25	-5.81	-117.01	-10.95	-5.45
6	-176.18	-23.36	-6.92	-179.44	-22.45	-6.83	-172.99	-15.34	-6.03	-172.38	-19.19	-6.51	-172.94	-19.86	-6.59	-166.17	-19.59	-6.61
7	-121.62	-14.47	-6.57	-130.00	-15.03	-6.63	-133.88	-15.91	-6.73	-138.81	-17.51	-6.91	-128.04	-13.31	-6.44	-122.01	-10.86	-6.18
8	-169.49	-19.93	-7.32	-171.46	-24.51	-7.73	-171.19	-21.96	-7.46	-176.43	-23.88	-7.67	-171.25	-18.03	-7.07	-158.99	-19.43	-7.17
9	-189.51	-16.91	-9.16	-184.75	-13.31	-8.52	-200.96	-16.05	-8.98	-202.19	-19.08	-9.34	-190.91	-13.75	-8.78	-173.53	-16.58	-8.93
10	-204.11	-12.24	-10.22	-224.22	-14.93	-10.82	-229.29	-15.30	-10.76	-229.41	-14.84	-10.88	-214.09	-9.42	-10.44	-206.84	-12.07	-10.74
11	-186.37	-14.89	-6.13	-190.37	-16.85	-6.36	-189.32	-18.46	-6.57	-202.74	-21.87	-6.92	-192.51	-19.31	-6.64	-184.02	-14.01	-6.03
12	-177.95	-19.71	-7.72	-180.85	-21.56	-7.92	-174.61	-23.88	-8.17	-176.75	-27.96	-8.59	-187.88	-18.55	-7.66	-176.19	-19.99	-7.75
13	-195.25	-29.08	-8.14	-199.79	-31.92	-7.13	-220.34	-35.02	-7.47	-211.43	-29.60	-7.08	-197.62	-24.29	-6.44	-200.15	-27.37	-8.14
14	-202.87	-24.00	-6.69	-208.91	-29.56	-6.75	-197.11	-22.86	-6.02	-211.30	-27.81	-6.52	-194.87	-20.23	-5.66	-191.92	-21.28	-5.78
15	-188.38	-20.16	-6.74	-191.83	-18.05	-6.46	-199.42	-18.33	-6.55	-204.04	-19.75	-6.71	-196.77	-19.93	-6.74	-181.46	-17.83	-6.40
16	-179.79	-17.39	-8.89	-172.27	-15.53	-8.64	-155.84	-13.49	-8.29	-166.38	-11.38	-8.06	-198.80	-23.24	-9.90	-164.07	-11.74	-8.08
17	-201.30	-20.30	-7.14	-193.04	-25.02	-7.70	-198.81	-21.80	-7.35	-200.17	-20.59	-7.23	-190.96	-21.62	-7.25	-177.92	-21.29	-7.24
18*	-190.80	-23.92	-6.63	-194.03	-26.76	-7.07	-201.01	-26.43	-6.87	-203.02	-27.79	-7.30	-189.45	-22.98	-6.38	-188.75	-25.73	-7.04
RMSD*	0.176			0.191			0.210			0.242			0.145			0.259		

*reference ligand: SAM or Sinefungin

Table S9. The physico-chemical parameters describing the studied ligands.

Ligand	MW	QED	Flexibility	Golden triangle	XLogP	Consensus LogP	Solubility (SILICOS-IT)	TPSA [A²]	SAS	CaCo-2	PAMPA	BBB
1	266.25	0.528	0.13	YES	-1.10	-1.02	-1.87	126.65	3.73	-5.88	0.9334	No
2	291.26	0.521	0.13	YES	-1.38	-1.22	-1.87	150.44	3.91	-5.89	0.9612	No
3	309.28	0.422	0.19	YES	-2.23	-1.77	-2.68	169.74	3.93	-5.78	0.9867	No
4	398.44	0.345	0.44	YES	-3.49	-2.96	-3.37	210.76	4.90	-6.08	0.9998	No
5	267.24	0.491	0.13	YES	-1.05	-1.57	-2.37	139.54	3.86	-5.90	0.9375	No
6	381.39	0.307	0.44	YES	-4.31	-2.84	-3.03	208.65	4.74	-6.04	0.9996	No
7	392.15	0.495	0.13	YES	-0.45	-0.36	-0.90	126.65	4.00	-5.58	0.9465	No
8	384.41	0.352	0.44	YES	-3.50	-2.22	-2.29	207.93	4.69	-5.93	0.9986	No
9	480.42	0.3	0.59	YES	0.15	-0.48	-1.27	211.01	4.78	-5.80	0.9229	No
10	605.49	0.17	0.78	NO	0.90	0.16	-0.73	203.09	5.41	-5.79	0.9214	No
11	459.39	0.17	0.42	YES	-5.94	-3.45	-4.64	272.63	5.03	-5.95	1.0000	No
12	414.44	0.317	0.50	YES	-3.48	-2.27	-2.22	217.16	4.94	-5.92	0.9885	No
13	414.44	0.097	0.74	NO	-3.48	-2.27	-2.22	217.16	4.94	-5.84	1.0000	No
14	466.49	0.098	0.65	YES	-5.30	-3.53	-3.84	250.27	5.11	-6.09	1.0000	No
15	440.52	0.302	0.69	YES	-1.59	-0.86	-0.68	193.94	5.11	-5.89	0.9999	No
16	496.58	0.263	0.55	YES	-2.51	-1.02	-1.17	223.98	5.66	-6.06	0.9973	No
17	456.52	0.188	0.69	YES	-3.50	-2.31	-2.59	245.98	5.21	-5.98	0.9998	No
New	413.15	0.202	0.56	YES	-2.80	-2.35	-0.52	219.96	4.93	-6.21	0.9993	No

Table S10. The pharmacokinetic and toxicity profiles of the studied ligands.

Lig-and	PAINS	BRENK	PGP substrate	PPB [%]	Carcinogenicity	Genotoxic carcionogenicty /mutagenicty	AMES	H-HT	DILI
1	0	0	0.38	31.72	0.68	0.99	0.95	0.83	0.96
2	0	0	0.01	50.31	0.64	0.99	0.90	0.87	0.97
3	0	0	0.67	24.60	0.67	1.00	0.95	0.92	0.99
4	0	1	0.99	18.14	0.70	1.00	0.96	0.87	0.73
5	0	0	0.39	36.87	0.65	0.99	0.91	0.81	0.95
6	0	0	0.70	15.07	0.31	0.99	0.67	0.75	0.60
7	0	1	0.05	45.90	0.62	0.99	0.80	0.79	0.78
8	0	0	0.14	26.06	0.67	1.00	0.92	0.60	0.54
9	0	0	0.05	63.57	0.57	1.00	0.64	0.71	0.94
10	0	1	0.85	62.81	0.50	1.00	0.37	0.81	0.20
11	0	2	0.00	22.07	0.39	1.00	0.89	0.95	1.00
12	0	0	0.14	48.76	0.76	0.89	0.82	0.45	0.16
13	0	0	0.00	15.15	0.84	1.00	0.32	0.08	0.95
14	0	2	1.00	10.14	0.12	1.00	0.93	0.59	0.42
15	0	0	0.10	44.26	0.46	0.82	0.65	0.42	0.20
16	0	0	0.99	15.08	0.96	0.81	0.72	0.80	0.28
17	0	0	0.99	13.40	0.53	0.93	0.54	0.40	0.02
New	0	0	0.56	22.37	0.39	1.00	0.22	0.15	0.96
